# TNFAIP2 promotes HIF1α transcription and breast cancer angiogenesis by activating the Rac1-ERK-AP1 signaling axis

**DOI:** 10.1038/s41419-024-07223-2

**Published:** 2024-11-13

**Authors:** Wenlong Ren, Huichun Liang, Jian Sun, Zhuo Cheng, Wenjing Liu, Yingying Wu, Yujie Shi, Zhongmei Zhou, Ceshi Chen

**Affiliations:** 1 School of Life Science, University of Science & Technology of China, Hefei, Anhui China; 2 Key Laboratory of Animal Models and Human Disease Mechanisms of the Chinese Academy of Sciences and Yunnan Province, Kunming Institute of Zoology, Chinese Academy of Sciences, Kunming, China; 3 Yunnan Key Laboratory of Breast Cancer Precision Medicine, Yunnan Cancer Hospital, The Third Affiliated Hospital of Kunming Medical University, Peking University Cancer Hospital Yunnan, Kunming, China; 4 Department of Pathology, The First Affiliated Hospital of Kunming Medical University, Kunming, Yunnan China; 5 Department of Pathology, Henan Provincial People’s Hospital, Zhengzhou University, Zhengzhou, Henan China; 6 The School of Continuing Education, Kunming Medical University, Kunming, China; 7 Yunnan Key Laboratory of Breast Cancer Precision Medicine, Academy of Biomedical Engineering, Kunming Medical University, Kunming, ChinaAcademy of Biomedical Engineering, Kunming Medical University, Kunming, China

**Keywords:** Tumour angiogenesis, Breast cancer, Oncogenes, Targeted therapies, RNAi

## Abstract

Angiogenesis is well known to play a critical role in breast cancer. We previously reported that TNFAIP2 activates Rac1 to promote triple-negative breast cancer (TNBC) cell proliferation, migration, and chemoresistance. However, the potential contribution of TNFAIP2 to tumor angiogenesis remains unknown. In this study, we demonstrated that TNFAIP2 promotes TNBC angiogenesis by activating the Rac1-ERK-AP1-HIF1α signaling axis. Under hypoxia, TNFAIP2 activates Rac1 and ERK sequentially. Following that, ERK activates the AP-1 (c-Jun/Fra1) transcription factor. By employing chromatin immunoprecipitation and luciferase reporter assays, we showed that AP-1 directly interacts with the *HIF1α* gene promoter, thereby enhancing its transcription. The combined application of ERK inhibitors, U0126 or trametinib, with the VEGFR inhibitor Apatinib, additively suppresses angiogenesis and tumor growth of HCC1806 in nude mice. These findings provide new therapeutic strategies for TNBC.

## Introduction

Breast cancer has become a prevalent malignancy that presents a substantial risk to women’s health, with its frequency on the rise [1]. Due to its notable heterogeneity, breast cancer is categorized into ER/PR-positive, HER2-positive, and triple-negative breast cancer (TNBC). Among these subtypes, individuals with TNBC exhibit a higher incidence of distant metastasis and a less favorable prognosis compared to those with other subtypes [2]. This subtype represents approximately 20% of all cases of breast cancer [3].

Angiogenesis is a complex physiological or pathological process characterized by the formation of new blood vessels from existing vascular beds, serving as a crucial step in the growth, invasion, and metastasis of solid tissues [4]. This process is regulated by various factors within the tumor microenvironment, with Vascular Endothelial Growth Factor A (VEGFA) originating from tumor cells playing a particularly significant role through paracrine signaling [5, 6]. Hypoxia inducible factor-1α (HIF1α) is a key driver of angiogenesis in solid tumors [7, 8]. The low-level expression of HIF1α under normoxic conditions can be attributed to its hydroxylation and subsequent rapid degradation through the ubiquitin-proteasome pathway [9]. HIF1α overactivation in hypoxic conditions is primarily achieved through post-transtional mechanisms, there are also studies showing that the transcription level of *HIF1α* mRNA can be regulated by various cytokines and growth factors under normoxic conditions, including LPS, TNFα, IL-1β and IFNα [10]. Additionally, there is evidence suggesting that the transcription factor NF-κB can interact with *HIF1α* gene promoter to enhance its transcription [11].

TNFAIP2 plays a significant role in tissue development, angiogenesis, inflammatory response, tumor growth and drug resistance [12]. Its high expression has been observed in a variety of tumor cells, including nasopharyngeal carcinoma [13], malignant glioma [14], urothelial carcinoma [15], esophageal squamous cell carcinoma [16], and TNBC [17], and is correlated with unfavorable clinical outcomes. Our previous studies [17, 18] showed that TNFAIP2, as a KLF5 downstream target protein, can activate Rac1, a member of the Rho small GTP enzyme family, to induce changes in the cytoskeleton, leading to the formation of filopodia and lamellipodia, ultimately promotes proliferation, adhesion, migration, and invasion of TNBC cells [17]. We also revealed that TNFAIP2 promotes DNA damage repair by activating Rac1, promoting DNA damage drug resistance in TNBC [18].

Rac1 has been reported to promote tumor angiogenesis through VEGF [19, 20]. Additionally, Rac1 has been implicated in the upregulation of HIF1α protein expression by downregulating p53 and VHLα [21]. Moreover, Rac1 has the ability to stimulate ERK1/2, which in turn promotes the phosphorylation of DNA damage response-related proteins ATM/ATR and CHK1/2 [22].

MAPK is a member of the evolutionarily conserved serine/threonine protein kinase family in eukaryotes [23]. Currently, the identified members of the MAPK family primarily consist of ERK1/2, p38, c-Jun amino terminal kinase JNK, and ERK5 [24, 25]. Among all MAPK family members, ERK1/2 are studied most thoroughly. ERK1/2 is highly expressed in breast cancer cells and correlates with poor prognosis in TNBC patients with TNBC [26]. Inhibition of ERK1/2 through knockdown or treatment with the ERK1/2 inhibitor U0126 has been shown to facilitate the ubiquitination degradation of c-Myc, resulting in the downregulation of transcription factor Fra1 expression and ultimately suppressing the metastasis of MDA-MB-231 and MDA-MB-468 cells [27]. Additionally, there is evidence suggesting that blockade of the ERK1/2 signaling can downregulate c-Jun expression and disrupt the formation of AP-1 heterodimers [28]. Furthermore, ERK1/2 has been implicated in mediating *HIF1α* transcription induction by LPS [29].

In this study, we found that TNFAIP2 is essential for *HIF1α* transcriptional expression, thereby facilitating breast cancer angiogenesis. TNFAIP2 increases *HIF1α* gene transcription via the Rac1-ERK-AP-1 signaling pathway. These findings highlight the role and functional mechanism of TNFAIP2 and propose TNFAIP2 as a potential therapeutic target for inhibiting angiogenesis in TNBC.

## Materials and methods

### Cell culture and treatment

The cells utilized in the experiments were sourced from the American Type Culture Collection (ATCC) in Manassas, Virginia, USA, and were authenticated using Short Tandem Repeat (STR) analysis. HCC1806 cells were cultured in RPMI 1640 medium supplemented with 5% fetal bovine serum (FBS). MDA-MB-468 and HEK293T cells were cultured in DMEM (Thermo Fisher, Grand Island, USA) with 5% FBS at 37 °C in a 5% CO_2_ environment. The compounds U0126 (Cat#HY-12031), Trametinib (Cat#HY-10999) and Apatinib (Cat#HY-13342A) were procured from MCE in New Jersey, USA.

### Stable overexpression of TNFAIP2 and Rac1/Rac1-P29S

The full-length TNFAIP2/Rac1/Rac1-P29S genes were cloned and subsequently subcloned into the pCDH lentiviral vector. The packaging plasmids (pMDLg/pRRE, pRSV-Rev, and pCMV-VSV-G) along with the pCDH-TNFAIP2/Rac1/Rac1-P29S expression plasmid were co-transfected into HEK293T cells to generate lentivirus. After 48 h of transfection, the lentivirus was harvested and used to infect HCC1806 cells. Subsequently, puromycin (2 μg/ml) was applied to select for drug-resistant cell populations.

### Stable knockdown of TNFAIP2 and Fra1

The pSIH1-H1-puro shRNA vector was utilized for the expression of TNFAIP2, Fra1, and luciferase (Luc) shRNAs. The shRNA target sequences used in this study are listed in Supplementary Table 1. HCC1806 and MDA-MB-468 cells were infected with lentivirus, followed by puromycin (2 μg/ml) selection of cell populations after 48 h. The knockdown efficiency was assessed through Western blotting analysis.

### RNA interference

The siRNA target sequences used in this study are listed in Supplementary Table 1. All siRNAs were synthesized by RiboBio (RiboBio, China) and transfected at a final concentration of 50 nM.

### Antibodies and western blotting (WB)

Cells were lysed in RIPA lysis buffer containing protease inhibitor, followed by mixing of samples with 1×SDS buffer at 98 °C for 10 min. The samples were then subjected to separation by SDS-PAGE and transferred onto PVDF membranes (Millipore, Germany). Subsequently, the membranes were blocked with 5% nonfat milk in PBS with 0.1% Tween 20, and incubated with primary antibodies overnight at 4 °C. This was followed by incubation with horseradish peroxidase-labeled secondary antibodies for 1 h at room temperature. Signal detection was achieved using enhanced chemiluminescence reagent (UE, S6009) and ImageQuant LAS4000 (GE, Germany). The anti-TNFAIP2 (sc-28318), anti-p-ERK1/2 (sc-7383), anti-ERK2 (sc-154), anti-c-Jun (sc-74543) and anti-Fra1 (sc-376148) antibodies were purchased from Santa Cruz Biotechnology (Santa Cruz, CA, USA). The anti-HIF1α (#36169), anti-AKT (#4685), anti-p-AKT (#4060) antibodies were purchased from CST(Boston, MA, USA). Anti-Rac1 (05–389) antibody was purchased from Millipore (Billerica, MA, USA). The anti-β-actin (A5441) antibody was purchased from Sigma‒Aldrich (St Louis, MO, USA). Anti-Flag (M185-3L) antibody was purchased from MBL(Nagoya, Japan).

### Reverse transcriptase-PCR and quantitative reverse transcriptase-PCR

RNA samples were extracted using the TRIzol reagent (15596-026; Invitrogen). followed by reverse transcriptions were performed using the HiScript II Q RT SuperMix for qPCR (+gDNA wiper) with gDNA eraser (R223-01, Vazyme, China). For Quantitative reverse transcriptase PCR, we used the Taq Pro Universal SYBR qPCR Master Mix (Q712-02; Vazyme). The primer sequences The primer sequences were listed in Supplementary Table 2.

### Cell migration assays

In order to assess the migration of primary human umbilical vein endothelial cells (HUVECs), a wound-healing assay was conducted. After 24 h of seeding, the supernatants of the HUVECs were removed and the cells were scratched and cultured with the conditioned medium for an additional 24 h. The closure of the wound was then observed and recorded using microscopy. Subsequently, the width of the gap in each image was quantitatively analyzed using Image J software.

### Tube formation assays

HUVECs (1.25 × 10^4^) in conditioned medium were seeded onto Matrigel (BD Biosciences)-coated μ-Slide angiogenesis (ibidi GmbH, Munich, Germany). After 6 h, images were captured using microscopy and analyzed using Image ProPlus 6.0 software to measure the total tube length.

### In vitro VEGFA quantification

Following the treatment with siRNAs, lentivirus or ERK inhibitors, HCC1806 cells were exposed to environment for 24 h. Subsequently, the supernatants were collected, and VEGFA levels were quantified utilizing the Quantikine VEGFA enzyme-linked immunosorbent assay (ELISA) kit (DVE00, R&D Systems, UK).

### Dual-luciferase assays

The proximal *HIF1α* gene promoters were amplified from normal human DNA and subsequently cloned into the pGL3-Basic vector (Promega). HEK293T or HCC1806 cells were then seeded at a density of 1.25 × 10^5^ cells/well in 12-well plates. The following day, the cells were transfected with the pGL3-HIF1α-promoter reporter plasmid (0.5 μg/well) and a pRL-β-actin internal control (25 ng/well) in triplicate. Luciferase activity was measured 48 h post-transfection using the dual-luciferase reporter assay system (Promega, Beijing, China).

### Chromatin immunoprecipitation assays

The chromatin immunoprecipitation assay was conducted on PCDH-3×Flag-TNFAIP2-overexpressing HCC1806 cells according to the protocol provided by Abcam (Cambridge, MA, USA). The diluted DNA-protein complex was incubated with anti-Flag antibody overnight at 4 °C in the presence of herring sperm DNA and protein A/G beads. Subsequently, chromosomal DNA was purified and subjected to PCR and qPCR analysis. The PCR and qPCR primers used for amplifying the region of interest (−500 to 100 from ATG) on the *HIF1α* gene promoter were listed in Supplementary Table 3.

### Tumorigenesis in BALB/c nude mice

The animal experiments conducted in this study were ethically approved by the Ethics Committee of the Kunming Institute of Zoology, Chinese Academy of Sciences. Female nude mice, approximately 6 weeks old, were procured from SJA Lab Animal Co. Ltd. (Changsha, China) and subjected to bilateral orthotopic fat pad injection of HCC1806 cells at a concentration of 1 × 10^6^ cells per spot. Once the tumor reached a volume of 50 mm^3^, the mice were randomly allocated into four groups, each comprising 8 or 6 mice, and were treated with Apatinib (50 mg/kg, i.g. qd), and/or U0126 (10 mg/kg, i.p. qod)/trametinib (1 mg/kg, i.p. qod), or an equal volume of 50% PEG300 plus 50% saline as a control. Tumor dimensions were measured every 2 days using a vernier caliper. The tumors were harvested for analysis on day 18 after tumor cell injection. The tumor volume was calculated by the formula: (π × length × width^2^)/6.

### Immunohistochemical staining

Paraffin-embedded clinical TNBC specimens were obtained from the Department of Pathology, Henan Provincial People’s Hospital, Zhengzhou University, China. Informed consent was obtained from all subjects. Two tissue microarrays containing 85 TNBC breast cancer tissues and 95 cancer-adjacent normal breast tissues were constructed. The xenograft tumor tissues were preserved in 3.7% formalin solution. For the immunohistochemistry (IHC) assay, the slides were deparaffinized, rehydrated, and subjected to heat treatment in a pressure cooker for 2.5 min in EDTA for antigen retrieval. Endogenous peroxidase activity was inactivated by adding an endogenous peroxidase blocker (OriGene, China) for 15 min at room temperature. Slides were incubated overnight at 4 °C with anti-CD31 (1:400, Abcam, ab28364) or anti-TNFAIP2 (1:200, Santa Cruz, sc-28318). After 12 h, the slides were washed three times with PBS and incubated with secondary antibodies (hypersensitive enzyme-labeled goat anti-mouse/rabbit IgG polymer (OriGene, China) at room temperature for 20 min, DAB concentrate chromogenic solution (1:200 dilution of concentrated DAB chromogenic solution), counterstained with 0.5% hematoxylin, dehydrated with graded concentrations of ethanol for 3 min each (70–80–90–100%), and finally cleared with dimethyl benzene. Immunostained slides were evaluated by light microscopy. The IHC signal was scored using the ‘Allred Score’ method.

### Statistical analysis

The graphs were generated using GraphPad Prism software version 8.0, and statistical analyses were conducted using SPSS 23 (SPSS Inc, USA). Each experiment was repeated a minimum of three times, with results presented as means ± standard deviation. Two-sided t-tests were utilized to compare differences between groups with similar variances. None of the samples were excluded. The researchers were not blinded during sample collection or data analysis. A significance level of *P* <0.05 was deemed statistically significant.

## Results

### TNFAIP2 promotes TNBC angiogenesis

**Fig. 1:**
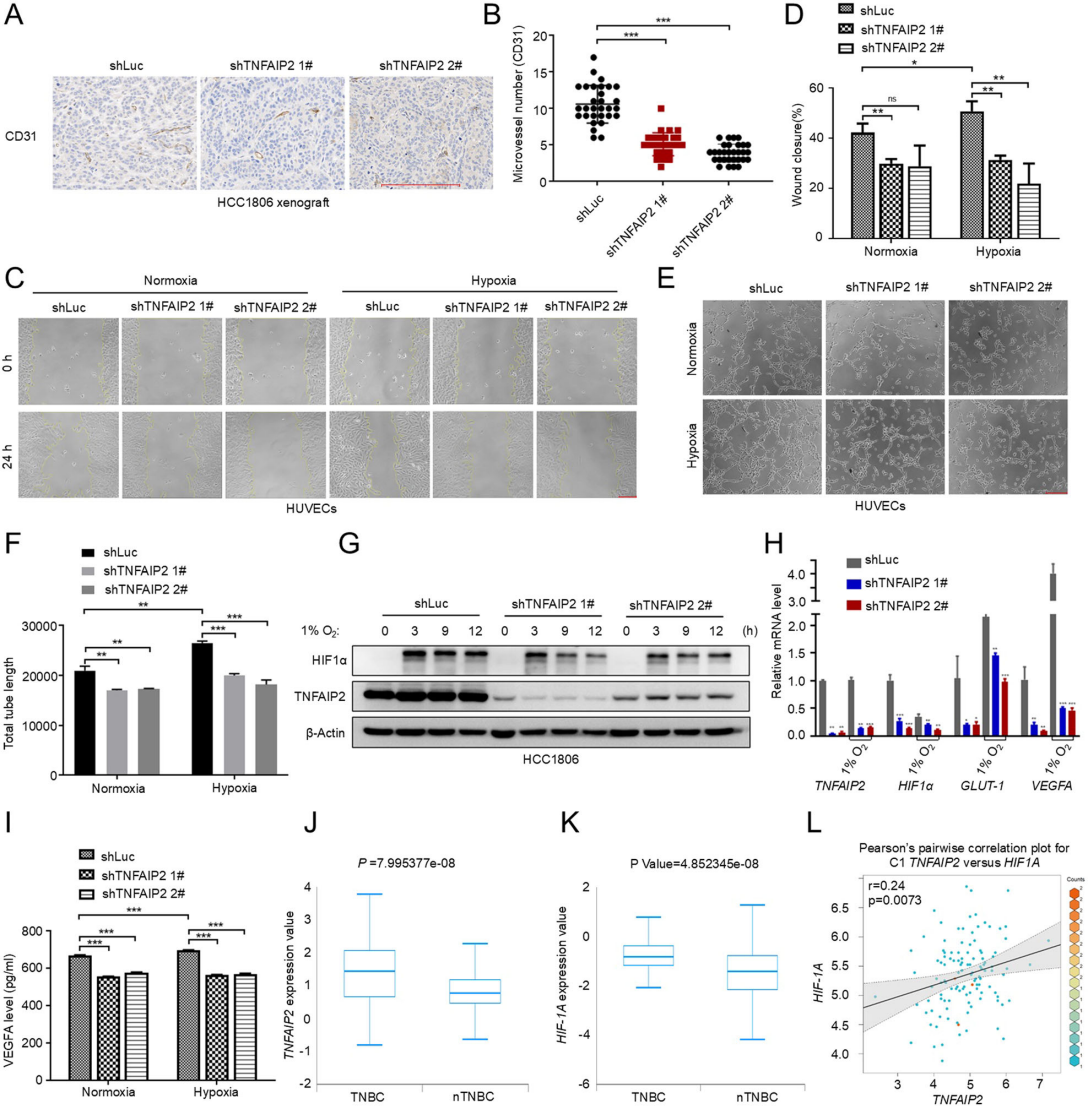
TNFAIP2 promotes angiogenesis and HIF1α expression in TNBC. **A**, **B** Knocking down TNFAIP2 in HCC1806 cells decreased the number of microvessels, as measured by the CD31 immunohistochemical staining. Represented images are shown. **C**, **D** Conditioned medium (CM) collected from TNFAIP2 knockdown HCC1806 cells failed to promote the migration of HUVECs compared to CM from control cells in the wound-healing assay. Representative images are shown. The cells were exposed to 1% O_2_ for 24 h. **E**, **F** The tube formation of HUVECs was inhibited by CM collected from TNFAIP2 knockdown HCC1806 cells. Representative images are shown. **G** Knockdown of TNFAIP2 in HCC1806 cells reduced hypoxia-induced HIF1α protein expression. The cells were exposed to 1% O_2_. **H** Knockdown of TNFAIP2 in HCC1806 cells reduced the mRNA levels of *HIF1α*, *GLUT-1* and *VEGFA*. The cells were exposed to 1% O_2_ for 48 h. Cell lysates were harvested for real-time PCR. **I** Knocking down TNFAIP2 in HCC1806 cells decreased the secreted VEGFA protein levels. The cells were exposed to 1% O_2_ for 24 h. **J**, **K** The expression levels of TNFAIP2 and HIF1α were elevated in TNBC patient samples compared to non-TNBC samples, based on TCGA database. **L** A positive correlation was observed between *TNFAIP2* and *HIF1α* mRNA levels in breast cancer patient samples, based on GEO database. Scale bar, 200 μm, in the histogram, the bars represent the mean ± SD (n = 3), *P < 0.05, **P < 0.01 and ***P < 0.001; ns not significant.

KLF5 has been shown to promote angiogenesis [30]. Our previous studies demonstrated that TNFAIP2 is a KLF5 downstream target gene in response to TNFα and TNFAIP2 knockdown inhibited HCC1806 xenograft tumor growth [17, 31]. To investigate whether TNFAIP2 promotes angiogenesis in TNBC, we examined CD31 expression in HCC1806 xenograft tumors using immunohistochemistry (IHC). The results revealed a significant decrease in CD31 staining in TNFAIP2-knockdown tumors compared to the control (Fig. 1A, B). Moreover, conditioned medium (CM) from breast cancer cell lines HCC1806 and MDA-MB-468 cultured under hypoxia was collected to stimulate HUVECs for wound healing and tube formation assays (Fig. S1A). The results demonstrated that CM under hypoxia significantly enhanced migration and tube formation of HUVECs compared to CM from normoxic conditions. Furthermore, knockdown of TNFAIP2 attenuated the proangiogenic effects induced by hypoxia in both breast cancer cell lines (Figs. 1C–F and S1B–E). These findings indicate that TNFAIP2 promotes hypoxia-induced angiogenesis in TNBC cells.

### TNFAIP2 promotes HIF1α expression in breast cancer cells

HIF1α is well documented to play a crucial role in angiogenesis [8]. To investigate whether TNFAIP2 promotes HIF1α expression in TNBC cells, we stably knocked down TNFAIP2 in HCC1806 and MDA-MB-468 cells and treated the cells with hypoxia. The results indicated that knockdown of TNFAIP2 significantly inhibited hypoxia-induced HIF1α protein expression in both cell lines (Figs. 1G and S1F). Moreover, the downregulation of TNFAIP2 resulted in a significant reduction in the mRNA levels of *HIF1α* and its downstream target genes, including *GLUT-1* and *VEGFA*, in both cell lines (Figs. 1H and S1G). Subsequently, CM were collected to assess the protein levels of VEGFA using ELISA. Consistent with the mRNA levels, knockdown of TNFAIP2 resulted in a reduction of VEGFA protein expression in HCC1806 cells (Fig. 1I). Furthermore, TNFAIP2 knockdown resulted in a reduction of *HIF1α* gene promoter activity (Fig. S1H).

To investigate the correlation between TNFAIP2 and HIF1α in TNBC, we analyzed data from The Cancer Genome Atlas (TCGA). A notable elevation of the expression levels of *TNFAIP2* and *HIF1α* in TNBC compared to non-TNBC tissues was observed (BCIP, Fig. 1J, K). Furthermore, examination of the Gene Expression Omnibus (GEO) database indicated a positive correlation between *TNFAIP2* and *HIF1α* expression in TNBC (GEPIA, Fig. 1L).

### TNFAIP2 promotes angiogenesis in vitro by upregulating the HIF1α expression

**Fig. 2:**
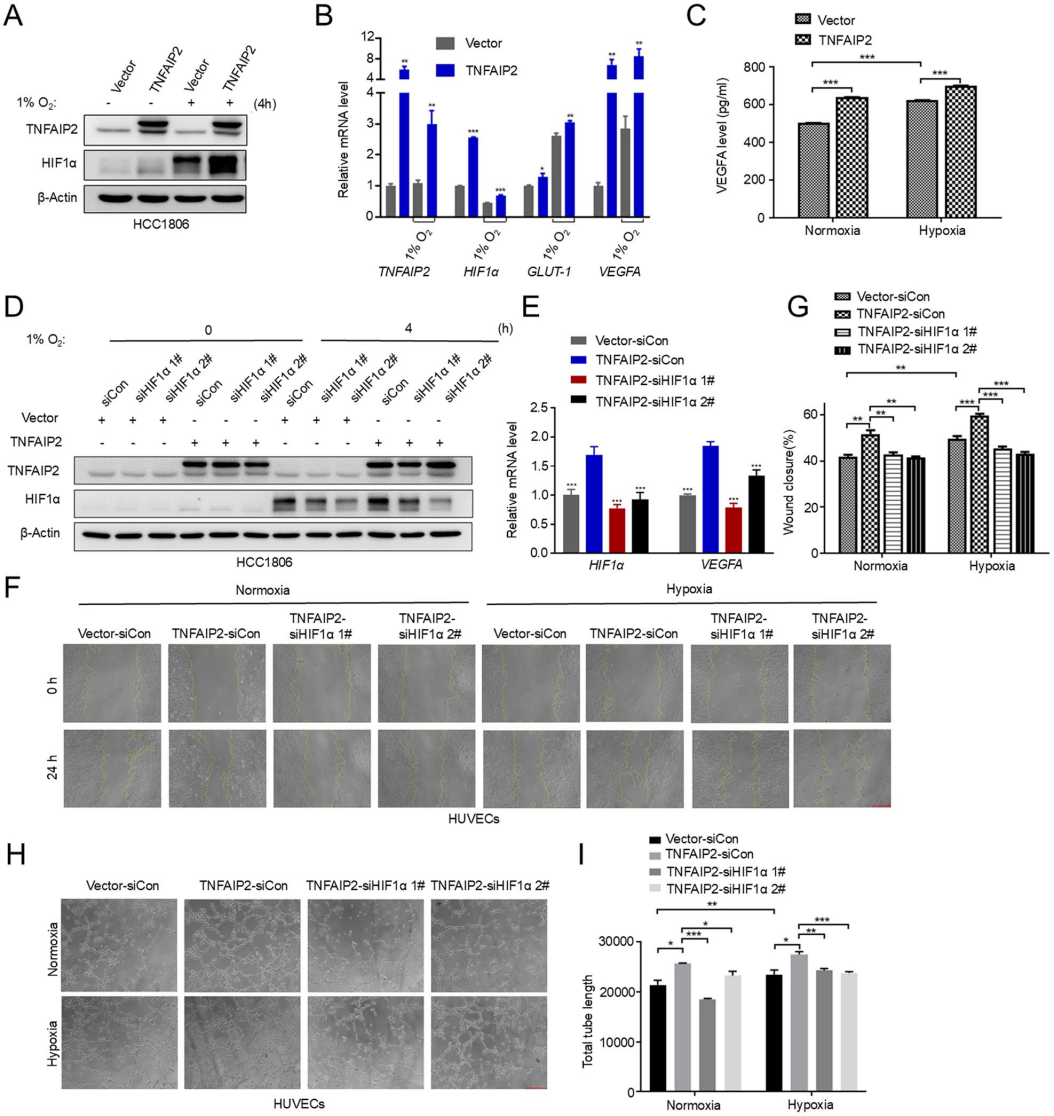
TNFAIP2 promotes angiogenesis in vitro by upregulating the HIF1α expression. **A** Overexpressing TNFAIP2 in HCC1806 cells increased HIF1α protein expression. Following stable overexpression of TNFAIP2, the cells were exposed to 1% O_2_. Cell lysates were harvested for WB analysis. **B** Overexpression of TNFAIP2 in HCC1806 cells increased the mRNA levels of *HIF1α*, *GLUT-1* and *VEGFA*. Following stable overexpression of TNFAIP2, the cells were exposed to 1% O_2_ for 48 h. Cell lysates were harvested for real-time PCR. **C** Overexpressing TNFAIP2 in HCC1806 cells increased the secreted VEGFA protein levels. The cells were exposed to 1% O_2_ for 24 h. **D** Overexpression of TNFAIP2 and knockdown of HIF1α in HCC1806 cells, as detected by WB. **E** HIF1α knockdown abolished TNFAIP2-induced *VEGFA* transcription upregulation. HCC1806 cells with stable TNFAIP2 overexpression were transfected with HIF1α siRNA. After 48 h of transfection, cell lysates were harvested for real-time PCR analysis. **F**, **G** Knockdown of HIF1α impeded the TNFAIP2 overexpression-induced migration of HUVECs by the wound-healing assay. Representative images are shown. **H**, **I** Knockdown of HIF1α impeded the TNFAIP2 overexpression-induced tube formation of HUVECs. Representative images are shown. Scale bar, 200 μm, in the histogram, the bars represent the mean ± SD (n = 3), *P < 0.05, **P < 0.01 and ***P < 0.001; ns not significant.

To investigate whether TNFAIP2 promotes the migration and tube formation of HUVECs by activating HIF1α/VEGFA signaling pathway, we knocked down HIF1α in TNFAIP2-overexpressing HCC1806 cells. The results suggest that the overexpression of TNFAIP2 upregulated *HIF1α*, *GLUT-1*, and *VEGFA* mRNA expression (Fig. 2A, B). Consistent with the mRNA levels, overexpression of TNFAIP2 resulted in a upregulation of VEGFA protein expression in HCC1806 cells (Fig. 2C), and the increase of *VEGFA* expression by TNFAIP2 was dependent on HIF1α (Fig. 2D, E). Moreover, depletion of HIF1α abrogated TNFAIP2 overexpression induced HUVEC migration and tube formation (Fig. 2F–I). These results provide evidence that TNFAIP2 promotes the migration and tube formation of HUVECs by upregulating the HIF1α expression.

### TNFAIP2 promotes *HIF1α* transcription and angiogenesis in vitro via Rac1

We previously reported that TNFAIP2 can activates Rac1 [17]. To test whether Rac1 promotes HIF1α expression, we knocked down Rac1 in HCC1806 and MDA-MB-468 cell lines and treated the cells with hypoxia. As expected, Rac1 knockdown caused a significant decrease expression of HIF1α and its downstream target genes *GLUT-1* and *VEGFA* in both cell lines (Fig. S2A–D). Consistent with the mRNA levels, knockdown of Rac1 resulted in a reduction of VEGFA protein expression in HCC1806 cells (Fig. S2E). Additionally, the hypoxia-induced proangiogenic effects in both breast cancer cell lines were attenuated by Rac1 knockdown (Fig. S2F–M). Moreover, we overexpressed Rac1-P29S, a constitutively activating mutant of Rac1 [32] in HCC1806 cells and found that Rac1-P29S enhanced the induction of HIF1α protein expression in HCC1806 cells by hypoxia (Fig. S2N). Subsequently, we knocked down HIF1α in Rac1-P29S-overexpressing HCC1806 cells and demonstrated that the induction of *VEGFA* by Rac1-P29S is hindered by the depletion of HIF1α (Fig. S2O, P). Consistently, the depletion of HIF1α abrogated Rac1-P29S overexpression induced HUVEC migration and tube formation (Fig. S2Q–T). Collectively, these findings support the notion that Rac1 facilitates the migration and tube formation of HUVECs through the upregulation of the HIF1α expression.

**Fig. 3:**
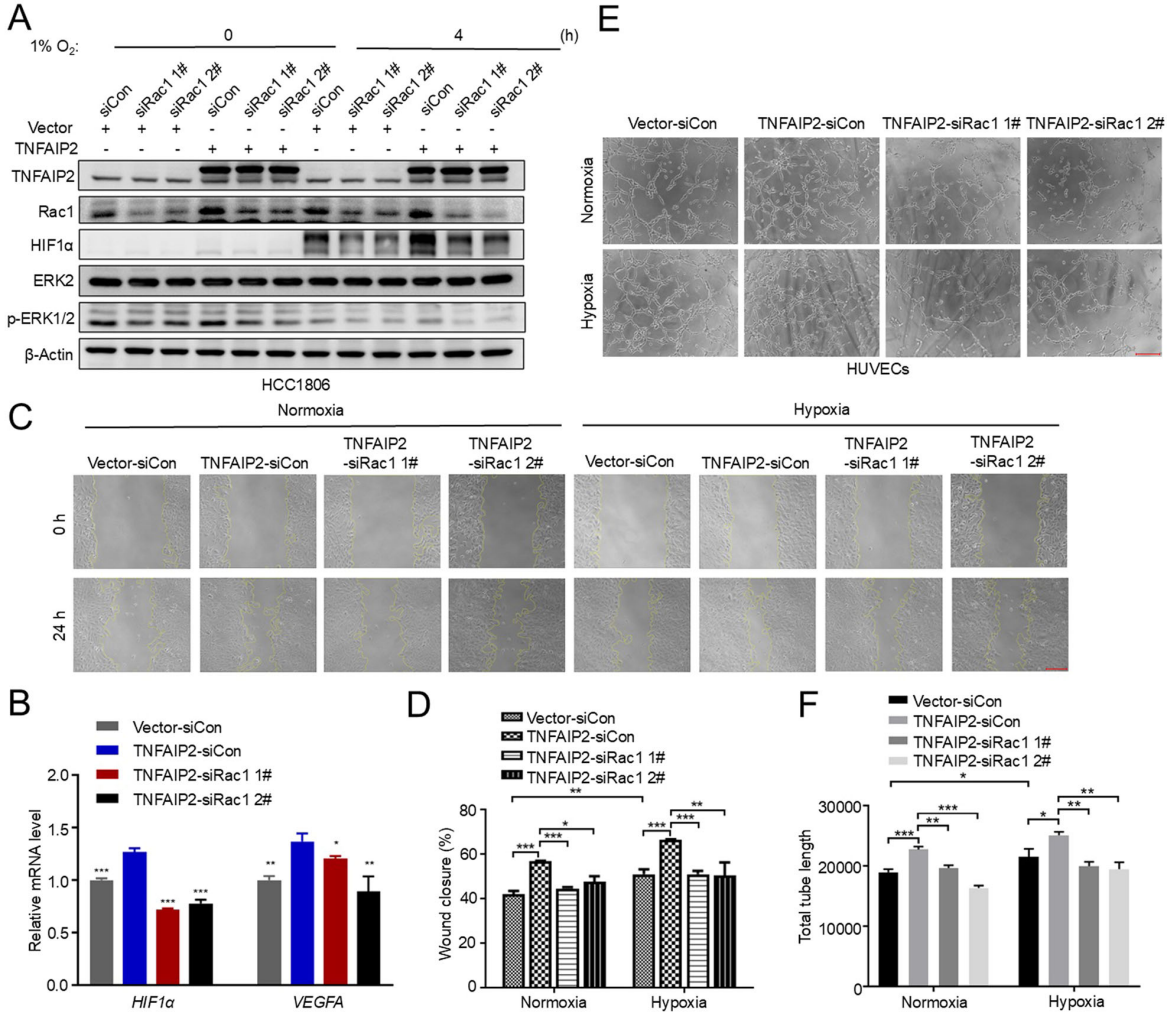
TNFAIP2 promotes *HIF1α* transcription and angiogenesis in vitro via Rac1. **A** Rac1 knockdown abolished TNFAIP2-induced HIF1α and p-ERK1/2 upregulation. HCC1806 cells with stable TNFAIP2 overexpression were transfected with Rac1 siRNA. After 24 h of transfection, the cells were exposed to 1% O_2_ for 4 h, cell lysates were harvested for WB analysis. **B** Rac1 knockdown abolished TNFAIP2-induced *HIF1α* and *VEGFA* mRNA upregulation. HCC1806 cells with stable TNFAIP2 overexpression were transfected with Rac1 siRNA. After 48 h of transfection, cell lysates were harvested for real-time PCR analysis. **C**, **D** Rac1 knockdown impeded TNFAIP2 overexpression induced migration of HUVECs by the wound-healing assay. Representative images are shown. **E**, **F** Rac1 knockdown impeded TNFAIP2 overexpression induced tube formation of HUVECs. Representative images are shown. Scale bar, 200 μm, in the histogram, the bars represent the mean ± SD (n = 3), *P < 0.05, **P < 0.01 and ***P < 0.001; ns not significant.

To test whether TNFAIP2 promotes *HIF1α* transcription and angiogenesis in vitro via Rac1, we knocked down Rac1 in TNFAIP2-overexpressing HCC1806 cells and demonstrated that the induction of HIF1α and VEGFA by TNFAIP2 is impeded by the knockdown of Rac1 (Fig. 3A, B). Consistently, the inhibition of Rac1 abrogated TNFAIP2 overexpression induced HUVEC migration and tube formation (Fig. 3C–F). These findings collectively support the notion that TNFAIP2 facilitates the migration and tube formation of HUVECs through Rac1.

### TNFAIP2 and Rac1 promote *HIF1α* transcription and angiogenesis in vitro via ERK

Next, we wondered how Rac1 promotes *HIF1α* transcription and angiogenesis. It has been documented that Rac1 can activate ERK1/2 [22], which in turn is responsible for the LPS-induced increase of *HIF1α* mRNA [29]. Therefore, we hypothesized that TNFAIP2 upregulates HIF1α expression through the Rac1-ERK pathway. To test this hypothesis, we knocked down TNFAIP2 or Rac1 and analyzed the ERK1/2 phosphorylation. As expected, knockdown of either TNFAIP2 or Rac1 resulted in a reduction in p-ERK1/2 levels (Figs. S3A, B and S4A, B). Moreover, U0126 significantly reduced the hypoxia-induced *HIF1α*, *GLUT-1* and *VEGFA* expression levels in HCC1806 and MDA-MB-468 cells (Fig. S3C–F). We further confirmed this result by ERK2 siRNAs (Fig. S3G–J). Moreover, the inhibition of ERK1/2 led to a decrease in the migratory and tube formation capabilities of HUVECs in both breast cancer cell lines (Fig. S3K–Z). These findings suggest that the ERK signaling pathway facilitates the migration and tube formation of HUVECs.

**Fig. 4:**
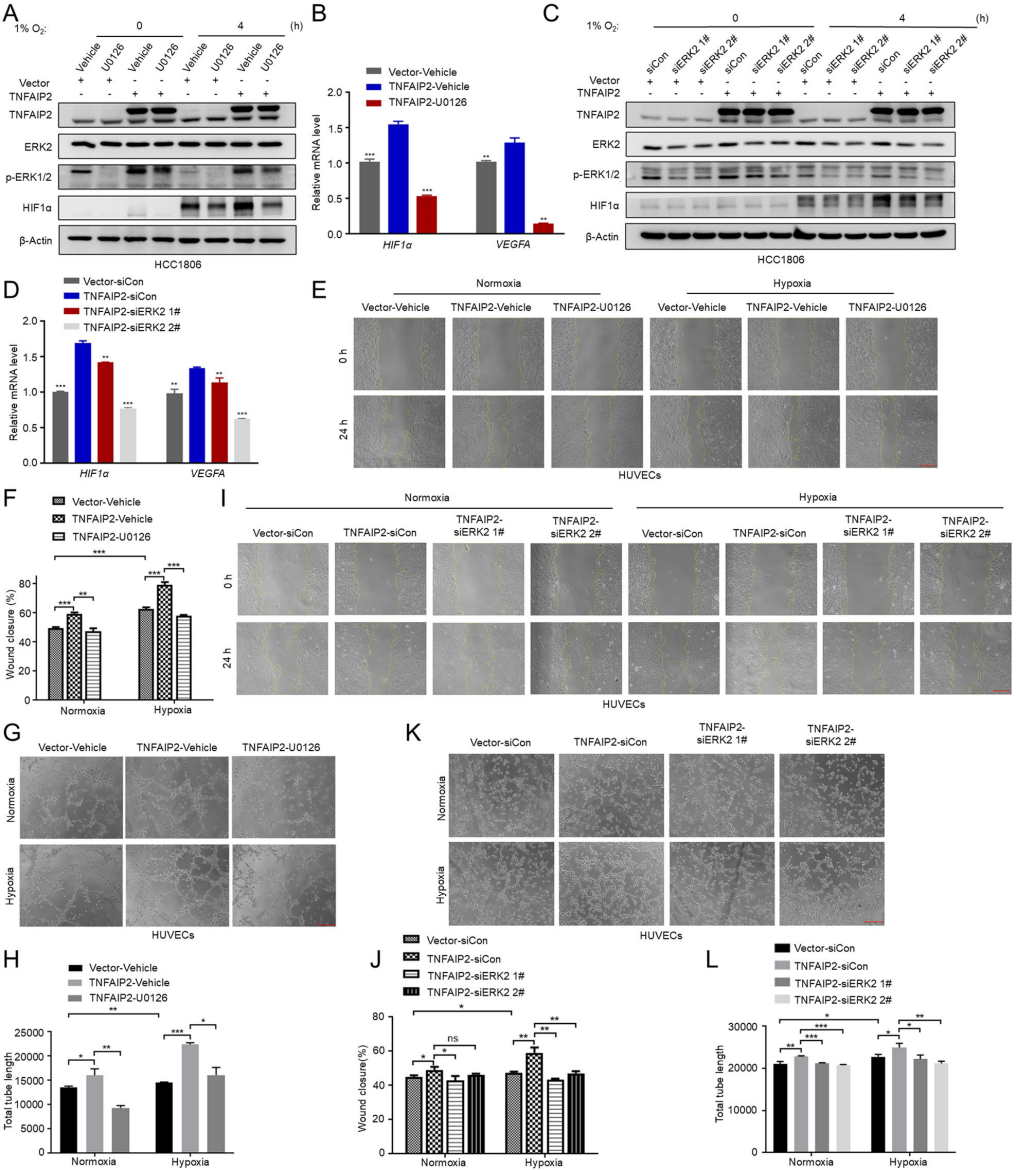
TNFAIP2 promotes *HIF1α* transcription and angiogenesis in vitro via ERK signaling. **A** ERK1/2 inhibition abolished TNFAIP2-induced HIF1α upregulation. HCC1806 cells with stable TNFAIP2 overexpression were treated U0126 (10 μM, 12 h) and exposed to 1% O_2_ for 4 h. Cell lysates were harvested for WB analysis. **B** ERK1/2 inhibition abolished TNFAIP2-induced *HIF1α* and *VEGFA* mRNA upregulation. HCC1806 cells with stable TNFAIP2 overexpression were treated U0126 (10 μM, 12 h). Cell lysates were harvested for real-time PCR analysis. **C** ERK2 knockdown abolished TNFAIP2-induced HIF1α upregulation in response to hypoxia. **D** ERK2 knockdown abolished TNFAIP2-induced HIF1α and VEGFA mRNA upregulation. **E**, **F** ERK1/2 inhibition impeded the TNFAIP2 overexpression-induced migration of HUVECs by the wound-healing assay. Representative images are shown. **G**, **H** ERK1/2 inhibition impeded the TNFAIP2 overexpression-induced tube formation of HUVECs. Representative images are shown. **I**, **J** ERK2 knockdown impeded the TNFAIP2 overexpression-induced migration of HUVECs by the wound-healing assay. Representative images are shown. **K**, **L** ERK2 knockdown impeded the TNFAIP2 overexpression-induced tube formation of HUVECs. Representative images are shown. Scale bar, 200 μm, in the histogram, the bars represent the mean ± SD (n = 3), *P < 0.05, **P < 0.01 and ***P < 0.001; ns not significant.

To test whether TNFAIP2 promotes HIF1α expression through ERK, we inhibited ERK signaling in TNFAIP2-overexpressing HCC1806 cells. The results indicated that the upregulation of *HIF1α* and *VEGFA* induced by TNFAIP2 was impeded by the inhibition of ERK signaling (Fig. 4A–F). Furthermore, inhibition of ERK-blocked TNFAIP2 overexpression induced HUVEC migration and tube formation (Fig. 4G–L). Moreover, when we treated Rac1-P29S-overexpressing HCC1806 cells with U0126 or ERK2 siRNA, Rac1-P29S induced HUVEC migration and tube formation were blocked (Fig. S4C–N). These findings collectively support the conclusion that TNFAIP2 and Rac1 facilitates the migration and tube formation of HUVECs through ERK.

### TNFAIP2 and Rac1 promote *HIF1α* transcription and angiogenesis in vitro via ERK-activated AP-1

**Fig. 5:**
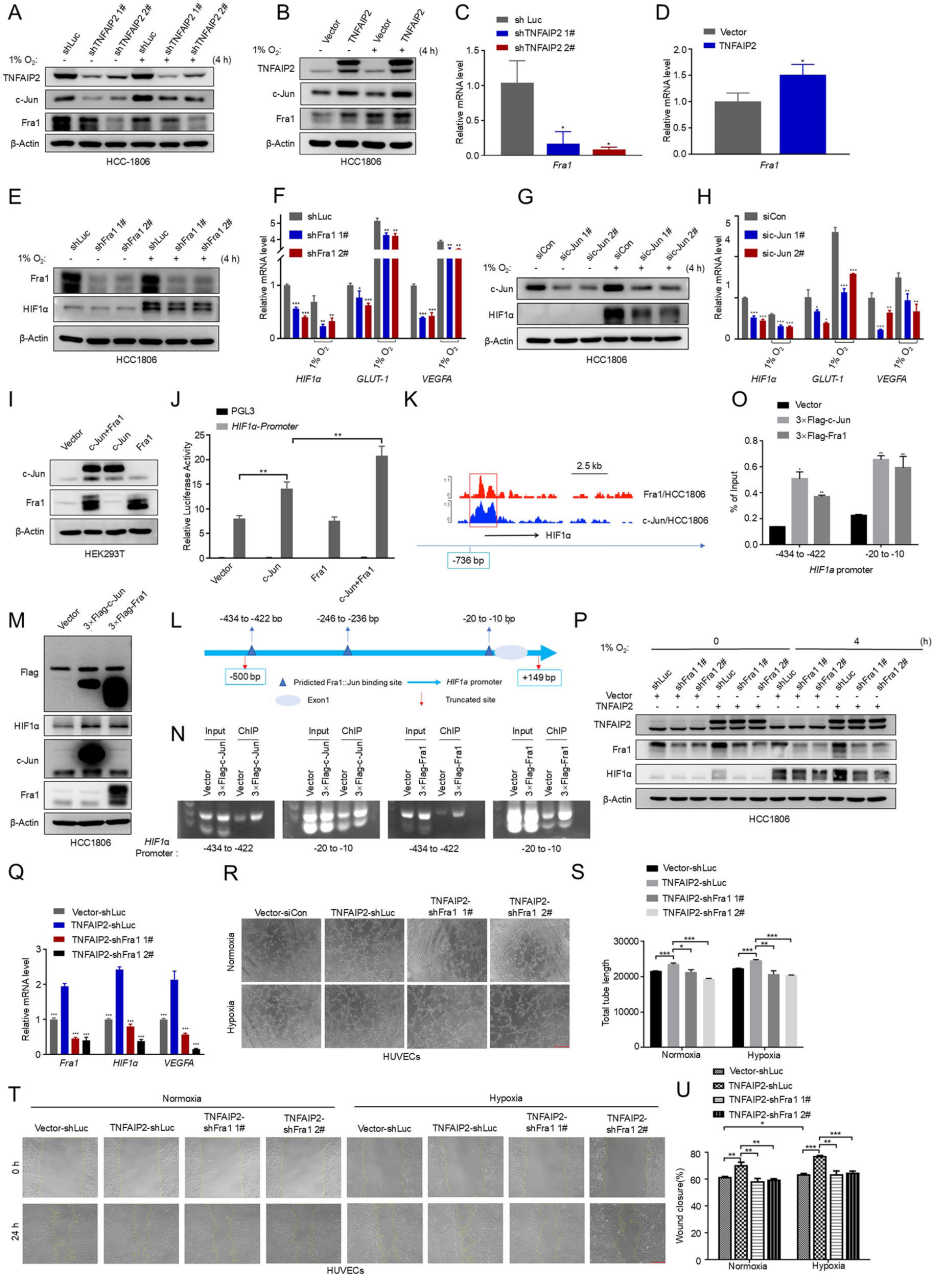
TNFAIP2 promotes *HIF1α* transcription and angiogenesis in vitro via AP-1. **A** Knockdown of TNFAIP2 in HCC1806 cells reduced the protein levels of c-Jun and Fra1. **B** Overexpression of TNFAIP2 in HCC1806 cells increased the protein levels of c-Jun and Fra1. **C** Knockdown of TNFAIP2 in HCC1806 cells reduced the mRNA levels of Fra1. **D** Overexpressing TNFAIP2 in HCC1806 cells increased the mRNA levels of Fra1. **E** Knockdown of Fra1 in HCC1806 cells reduced hypoxia-induced HIF1α protein expression. HCC1806 cells with stable Fra1 knockdown were exposed to 1% O_2_ for 4 h. Cell lysates were harvested for WB analysis. **F** Knockdown of Fra1 in HCC1806 cells reduced the mRNA levels of *HIF1α*, *GLUT-1* and *VEGFA*. Following stable knockdown of Fra1, the cells were exposed to 1% O_2_ for 48 h, and cell lysates were harvested for real-time PCR analysis. **G** Knockdown of c-Jun in HCC1806 cells reduced hypoxia-induced HIF1α protein expression. **H** Knockdown of c-Jun in HCC1806 cells reduced the mRNA levels of *HIF1α*, *GLUT-1* and *VEGFA*. **I** Overexpression of c-Jun and Fra1 in HEK293T cells **J** The *HIF1α* gene promoter was significantly activated by co-overexpression of c-Jun and Fra1, as determined by dual-luciferase assays. **K** Endogenous c-Jun and Fra1 binds to the *HIF1α* gene promoter as determined by Chromatin immunoprecipitation followed by sequencing (ChIP-seq) analysis. **L** Schematic of the predicted AP-1 binding sites at the *HIF1α* gene promoter (−500 to +100 bp). **M** Stable overexpression of c-Jun and Fra1 in HEK293T cells. **N**, **O** Binding of c-Jun and Fra1 to the *HIF1α* gene promoter was determined by ChIP-PCR and ChIP-qPCR in c-Jun or Fra1-overexpression HCC1806 cells. **P** Fra1 knockdown abolished TNFAIP2-induced HIF1α upregulation. HCC1806 cells with stable TNFAIP2 overexpression and Fra1 knockdown were exposed to 1% O_2_ for 4 h, and cell lysates were harvested for WB analysis. **Q** Fra1 knockdown abolished TNFAIP2-induced *HIF1α* and *VEGFA* mRNA upregulation. Cell lysates of HCC1806 cells with stable TNFAIP2 overexpression and Fra1 knockdown were harvested for real-time PCR analysis. **R**, **S** Fra1 knockdown impeded the TNFAIP2 overexpression-induced tube formation of HUVECs. Representative images are shown. **T**, **U** Fra1 knockdown impeded the TNFAIP2 overexpression-induced migration of HUVECs by the wound-healing assay. Representative images are shown. Scale bar, 200 μm, in the histogram, the bars represent the mean ± SD (n = 3), *P < 0.05, **P < 0.01 and ***P < 0.001; ns not significant.

AP-1 transcription factor is a dimeric complex consisting of various members from the JUN (c-Jun, Jun-B, Jun-D) and FOS (c-Fos, Fra-1, Fra-2, and Fos-B) protein families. Specifically, c-Jun exhibits the highest transcriptional activity within the Jun subfamily, while the Fos subfamily member Fra1 is prominently expressed in TNBC cells and is linked to unfavorable prognostic outcomes [33–35]. Moreover, c-Jun was reported to stimulate HIF1α *mRNA* expression in response to LPS [36].To verify whether TNFAIP2 regulates the transcription of *HIF1α* through AP-1, we assessed c-Jun and Fra1 levels following TNFAIP2 manipulation in HCC1806 cells. The results suggest that TNFAIP2 could upregulate the expression of c-Jun and Fra1 (Fig. 5A–D). Moreover, inhibition of ERK and Rac1 downregulated the expression of c-Jun and Fra1 in HCC1806 cells (Fig. S5A–F). Knockdown of c-Jun and Fra1 attenuated the hypoxia-induced upregulation of *HIF1α*, *GLUT-1* and *VEGFA* expression in HCC1806 cells (Fig. 5E–H). Additionally, luciferase reporter assays illustrated that overexpression of c-Jun and Fra1 augmented the activity of the *HIF1α* promoter (Fig. 5I, J). Furthermore, ChIP-Seq data in HCC1806 cells indicates the direct binding of c-Jun and Fra1 to the *HIF1α* promoter (Fig. 5K). We validated this result by ChIP-PCR and ChIP-qPCR assays (Fig. 5L–O).

To test whether TNFAIP2 promotes *HIF1α* transcription through AP-1, we stably silenced Fra1 in TNFAIP2-overexpressing HCC1806 cells. The results demonstrated that the TNFAIP2 induced expression of *HIF1α*, *GLUT-1* and *VEGFA* was hindered by the knockdown of Fra1 (Fig. 5P, Q). Additionally, Silence of Fra1 blocked TNFAIP2 overexpression induced migration and tube formation of HUVECs (Fig. 5R–U).

### ERK and VEGFR inhibitors in combination inhibit TNBC tumor growth in vivo

**Fig. 6:**
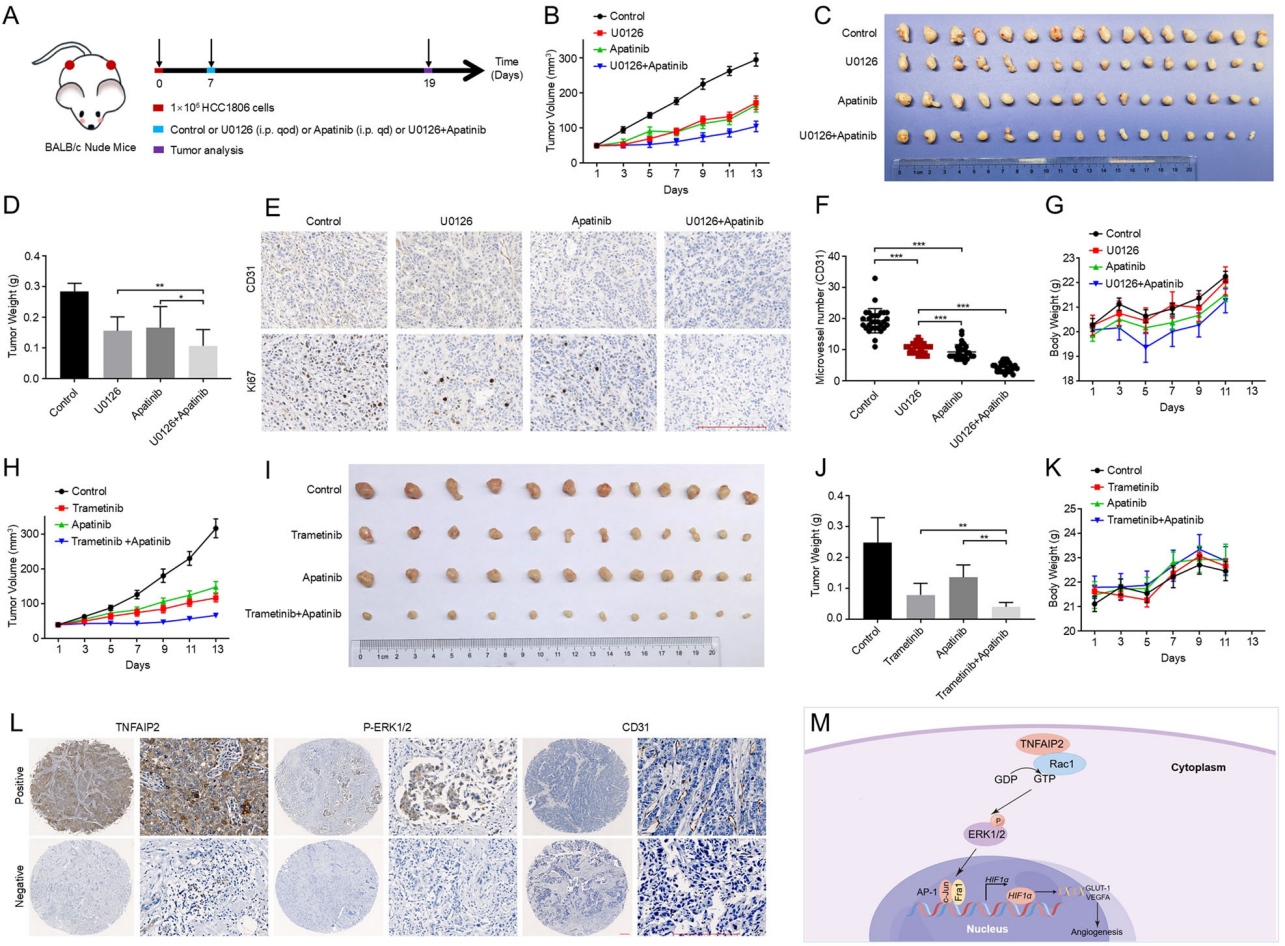
Apatinib and U0126 in combination treatment inhibits TNBC tumor growth in vivo*.* **A** Schematic diagram of orthotopic tumor model construction in nude mice and administration time and frequency. **B** Growth curves of xenograft tumors after the orthotopic injection of HCC1806 cells. Tumor growth was measured every 2 days. **C**, **D** Representative images and weights of tumors excised from mice after inoculation. All tumors were collected and weighed on the last day of the experiment. Data were represented as the mean ± s.d. of eight mice for each group (16 tumors). **E**, **F** The number of microvessels in tumors, as measured by the CD31 IHC. **G** Body weight of the mice. **H** Growth curves of xenograft tumors after the orthotopic injection of HCC1806 cells. Tumor growth was measured every 2 days. **I**, **J** Representative images and weights of tumors excised from mice after inoculation. All tumors were collected and weighed on the last day of the experiment. Data were represented as the mean ± s.d. of six mice for each group (12 tumors). **K** Body weight of the mice. **L** Representative IHC images of TNFAIP2, p-ERK and CD31 protein expression in TNBC tissues are shown. The final expression assessment was performed by combining the two scores (0 = Negative, 1–3 = Positive). **M** The work model of this study. Scale bar, 200 μm. *P < 0.05, **P < 0.01 and ***P < 0.001; ns not significant.

Since the TNFAIP2/Rac1/ERK/AP-1 axis promotes HIF1α-VEGFA expression and angiogenesis, we wondered how to translate our finding into the clinic. We explored the combination of U0126 with Apatinib in HCC1806 tumors in nude mice (Fig. 6A). The results indicate that U0126 and Apatinib, either alone or in combination, significantly inhibited tumor growth (Fig. 6B–D). Moreover, the tumor volume was significantly smaller in the combination group compared to the U0126 or Apatinib groups. Additionally, the number of microvessel in the xenograft tumors of the combination group was significantly lower than that in the U0126 or Apatinib groups, as determined by CD31 IHC staining (Fig. 6E, F). Importantly, the administration of U0126 and Apatinib showed minimal effects on the body weight of nude mice (Fig. 6G). Moreover, we probed the synergistic effects of combining Apatinib with the clinically approved ERK inhibitor trametinib (Fig. S5G, H). The results indicate that the tumor volume was significantly smaller in the combination group compared to the trametinib or Apatinib groups (Fig. 6H–J), and the administration of trametinib and Apatinib showed minimal effects on the body weight of nude mice (Fig. 6K). These findings indicate the antitumor efficacy of Apatinib in combination with U0126 or trametinib.

To test whether TNFAIP2 promotes TNBC angiogenesis, two breast cancer tissue chips containing 85 TNBC tissues and 95 cancer-adjacent normal breast tissues were collected and subjected to IHC analyses (Fig. 6L). The results indicate that TNFAIP2 protein expression levels were significantly positively correlated with p-ERK1/2 and CD31 (Table 1).

**Table 1:** TNFAIP2 expression levels positively correlated with p-ERK1/2 and CD31 in human TNBC specimens.

TNFAIP2									
R = 0.290^**^ P <0.01		Negative	Positive	Total	R = 0.370^**^ P <0.01		Negative	Positive	Total
**p-ERK1/2**	Negative	38	39	77	**CD31**	Negative	15	4	19
	Positive	0	8	8		Positive	23	43	66
	Total	38	47	85		Total	38	47	85

## Discussion

TNFAIP2 was initially characterized as a primary response gene in TNFα‐treated HUVECs, demonstrating its significance as an angiogenic factor with enhanced expression during the development of capillary tube‐like structures [31]. Furthermore, TNFAIP2 expression is associated with the intratumoral microvessel density in nasopharyngeal carcinomas [13]. In this study, we first found that TNFAIP2 promotes angiogenesis in TNBC through increasing *HIF1α* gene transcription. Next, we verified that TNFAIP2 promotes *HIF1α* transcription and TNBC angiogenesis by activating the Rac1-ERK-AP1 signaling axis. Finally, we demonstrated that the combination of U0126 or trametinib and Apatinib had additive antitumor effect.

Breast cancer has become a prevalent malignancy that presents a substantial risk to women’s health [1]. Metastatic breast cancer is a leading contributor to morbidity and mortality in patients with breast cancer [37, 38]. It is estimated that 20-30% of individuals initially diagnosed with early-stage breast cancer will ultimately develop metastatic disease, with angiogenesis playing a crucial role in the early stages of cancer metastasis [39]. Angiogenesis, the physiological process in which new blood vessels arise from existing ones, plays a crucial role in ensuring the provision of oxygen and nutrients required for tumor growth and metastatic dissemination to distant organs [40, 41]. Studies have shown that low-dose anti-angiogenic therapy could sensitize breast cancer to PD-1 blockade [42]. Furthermore, the combination of the PD-1 inhibitor camrelizumab with Apatinib showed a promising efficacy in the treatment of advanced TNBC [43]. Hence, further investigation into the angiogenesis mechanism of breast cancer is anticipated to yield a viable treatment approach for the disease, ultimately improving the survival rate of breast cancer patients.

As is well known, HIF1α plays a crucial role in regulating the adaptive responses of tumor cells to hypoxic conditions, its high expression has been observed in various types of tumors including breast cancer, and is associated with a poor prognosis [41]. HIF1α can transcriptionally activate several proangiogenic factors, such as VEGFA, which promotes the formation of new blood vessels to enhance oxygen delivery to tumor cells and facilitate their growth [44]. In normoxic conditions, HIF1α undergoes rapid degradation with a half-life of approximately 5 min [45]. Conversely, HIF1α is subject to regulation through various post-translational modifications including hydroxylation, acetylation, ubiquitination, and phosphorylation under hypoxic conditions, which subsequently impact its protein stability and transcriptional activity [46]. In the present study, we observed that the HIF1α protein level was increased in response to hypoxia (Fig. 1G). Conversely, a significant reduction in the *HIF1α* mRNA level was observed in breast cancer cells exposed to hypoxic conditions (Fig. 1F). This observation aligns with findings from our prior research [47]. Nevertheless, the specific mechanism responsible for the hypoxia-induced decline in *HIF1α* mRNA levels remains to be elucidated.

In this study, the results of luciferase reporter and ChIP assays demonstrated that AP-1(c-Jun/Fra1) promoted *HIF1α* transcriptional activity via directly binding to *HIF1α* gene promoter (Fig. 5A–M). Studies have shown that ERK can influence *Fra1* transcription through c-Myc in the MDA-MB-468 cells [27]. Indeed, we found that c-Myc is involved in regulating *Fra1* transcription by TNFAIP2 and ERK, ultimately leading to the promotion of *HIF1α* transcription (data not shown). Our previous works have indicated that TNFAIP2, a downstream target protein of KLF5, activates Rac1 to enhance the migration, invasion, and drug resistance of TNBC cells [15–17]. Building upon these findings, our research has demonstrated that TNFAIP2 can induce the transcription of *HIF1α* via the Rac1-ERK-AP1 signaling pathway, thereby promoting tumor angiogenesis in breast cancer. In future studies, it will be essential to generate *Tnfaip2* knockout mice in order to clarify the exact physiological role of TNFAIP2.

In spite of the increasing number of FDA-approved angiogenic agents, the long-term effectiveness of anti-angiogenic monotherapies is constrained [41]. Furthermore, the combined use of anti-angiogenesis agents alongside chemotherapy or immunotherapy shows potential as a strategy for cancer treatment [48]. Clinical studies have shown that the concurrent administration of the VEGF inhibitor bevacizumab with conventional chemotherapy leads to improved survival and response rates in patients with gastrointestinal cancer, non-small cell lung cancer, and breast cancer [49–51]. Additionally, studies have shown that Apatinib could enhance the sensitivity of TNBC cells to doxorubicin and paclitaxel [52, 53]. At present, there are no inhibitors available for TNFAIP2, and none of the inhibitors targeting Rac1 or HIF1α have received clinical approval. Conversely, inhibitors targeting MEK/ERK and VEGFA/VEGFR have been clinically approved. Consequently, an investigation was conducted to assess the potential of U0126 and trametinib in sensitizing Apatinib for the treatment of breast cancer in vivo. In this investigation, we demonstrated that U0126 and trametinib could further augment the antitumor efficacy of Apatinib in TNBC cells.

In conclusion, our research elucidated the role of TNFAIP2 in promoting angiogenesis in breast cancer. TNFAIP2 promotes breast cancer angiogenesis via the Rac1-ERK-AP1-HIF1α axis. These findings indicate that TNFAIP2 could be a promising therapeutic target for TNBC. In the meantime, ERK inhibitor in combination with VEGFR inhibitor is a potential treatment for TNBC.

## Supplementary information

Supplementary Material

Original western blots

## Data Availability

The authors confirm that the data supporting the findings of this study are available within the article.
