# Supplementary material for: TNFAIP2 promotes HIF1α transcription and breast cancer angiogenesis by activating the Rac1-ERK-AP1 signaling axis
Source: Cell Death Dis. 2024 Nov 13;15(11):821. doi: 10.1038/s41419-024-07223-2 (PMC11557851; doi:10.1038/s41419-024-07223-2)
Supplement: Supplementary file 1 — Supplementary Material [file 41419_2024_7223_MOESM1_ESM.docx]

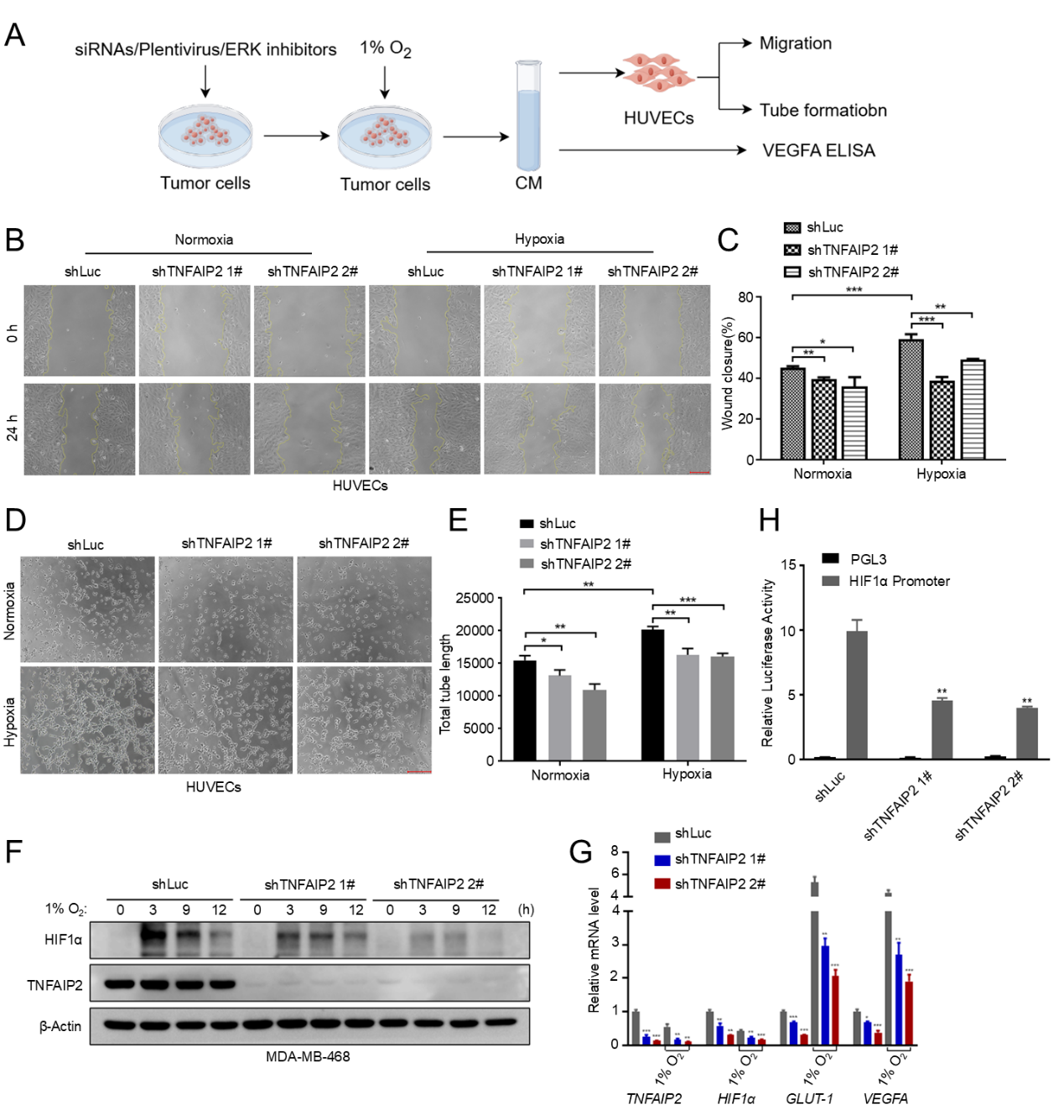
 **Figure S1. TNFAIP2 promotes TNBC angiogenesis *in vitro***

(A) Schematic illustration of the sample treatment and conditional medium (CM) collection. The CM was harvested for culturing the HUVECs. (B, C) The hypoxia-induced migration of HUVECs was inhibited by CM collected from TNFAIP2 knockdown MDA-MB-468 cells in the wound-healing assay. Representative images are shown. (D, E) The hypoxia-induced tube formation of HUVECs was inhibited by CM collected from TNFAIP2 knockdown MDA-MB-468 cells. Representative images are shown. (F) Knockdown of TNFAIP2 in MDA-MB-468 cells reduced HIF1α protein expression. (G) Knockdown of TNFAIP2 in MDA-MB-468 cells reduced the mRNA levels of *HIF1α*, *GLUT-1* and *VEGFA*. (H) Knockdown of TNFAIP2 in HCC1806 cells suppressed the luciferase activity of the *HIF1α* gene promoter. Following stable knockdown of TNFAIP2, the cells were transfected with the pGL3-Basic or pMIR-HIF1α-promoter together with pCMV-Renilla control. After 48 hours of transfection, cell lysates were harvested for dual-luciferase reporter assays.* *P* < 0.05, ** *P* < 0.01 and *** *P* < 0.001; ns, not significant.


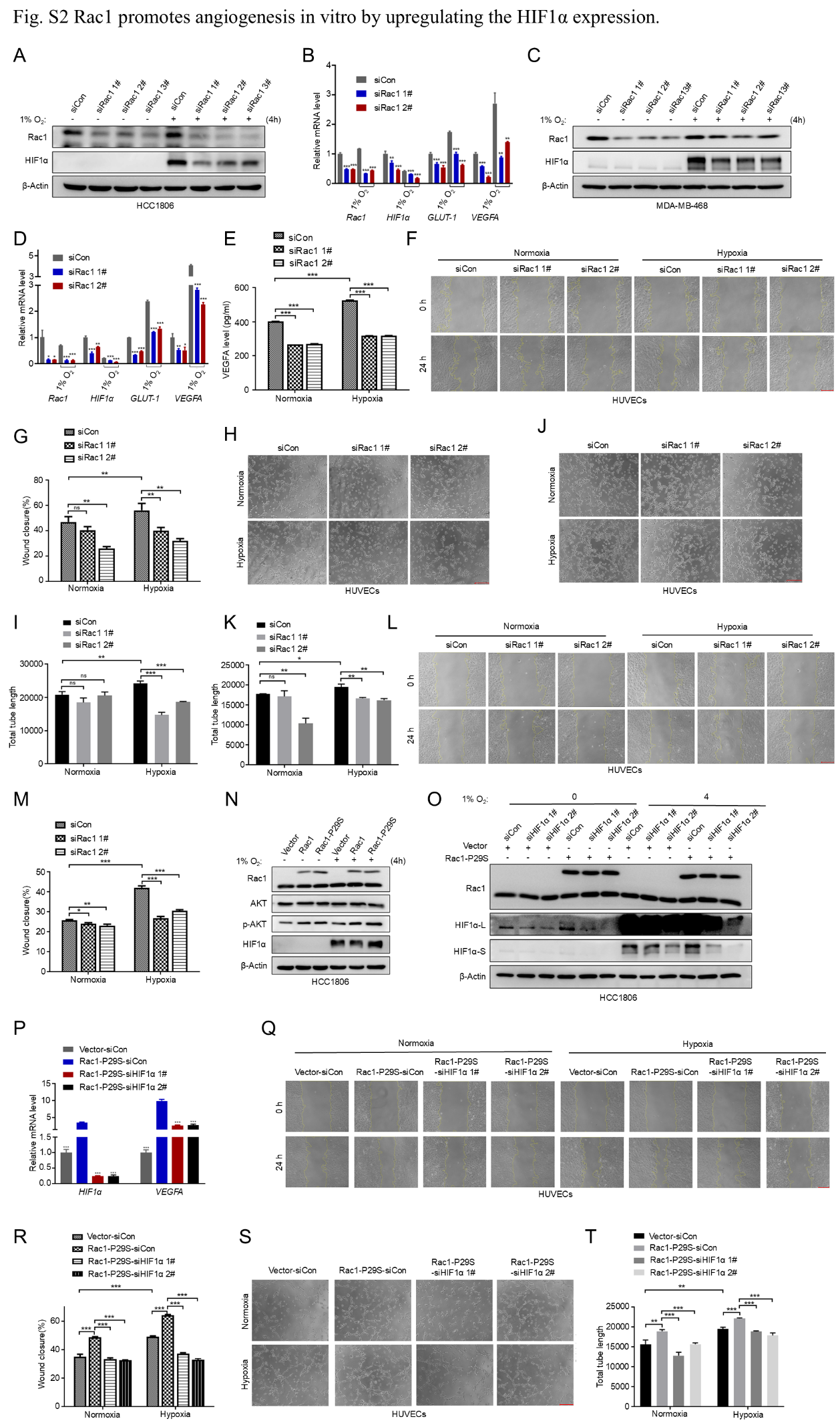
 **Figure S2.** **Rac1 promotes angiogenesis *in vitro* by upregulating the HIF1α expression**

(A) Knockdown of Rac1 in HCC1806 cells reduced HIF1α protein expression. (B) Knockdown of Rac1 in HCC1806 cells reduced the mRNA levels of *HIF1α*, *GLUT-1 a*nd *VEGFA.* (C) Knockdown of Rac1 in MDA-MB-468 cells reduced HIF1α protein expression. (D) Knockdown of Rac1 in MDA-MB-468 cells reduced the mRNA levels of *HIF1α*, *GLUT-1* and *VEGFA*. (E) Knocking down Rac1 in HCC1806 cells decreased the secreted VEGFA protein levels. The cells were exposed to 1% O_2_ for 24 hours. (F, G) The hypoxia-induced migration of HUVECs was inhibited by CM collected from Rac1 knockdown HCC1806 cells in the wound-healing assay. Representative images are shown. (H, I) The hypoxia-induced tube formation of HUVECs was inhibited by CM collected from Rac1 knockdown HCC1806 cells. Representative images are shown. (J, K) The hypoxia-induced migration of HUVECs was inhibited by CM collected from Rac1 knockdown MDA-MB-468 cells in the wound-healing assay. Representative images are shown. (L, M) The hypoxia-induced tube formation of HUVECs was inhibited by CM collected from Rac1 knockdown MDA-MB-468 cells. Representative images are shown. (N) The overexpression of Rac1-P29S (an activative mutant of Rac1) increase p-AKT and hypoxia-induced HIF1α protein expression in HCC1806 cells. (O) Overexpression of Rac1-P29S and knockdown of HIF1α in the HCC1806 cells, as detected by WB. (P) HIF1α knockdown abolished Rac1-P29S-induced VEGFA mRNA upregulation. HCC1806 cells with stable TNFAIP2 overexpression were transfected with HIF1α siRNA. After 48 hours of transfection, cell lysates were harvested for real-time PCR analysis. (Q, R) Knockdown of HIF1α impeded the Rac1-P29S overexpression induced migration of HUVECs in the wound-healing assay. Representative images are shown. (S, T) Knockdown of HIF1α impeded the Rac1-P29S overexpression induced tube formation of HUVECs. Representative images are shown. Scale bar, 200 μm.* *P* < 0.05, ** *P* < 0.01 and *** *P* < 0.001; ns, not significant.


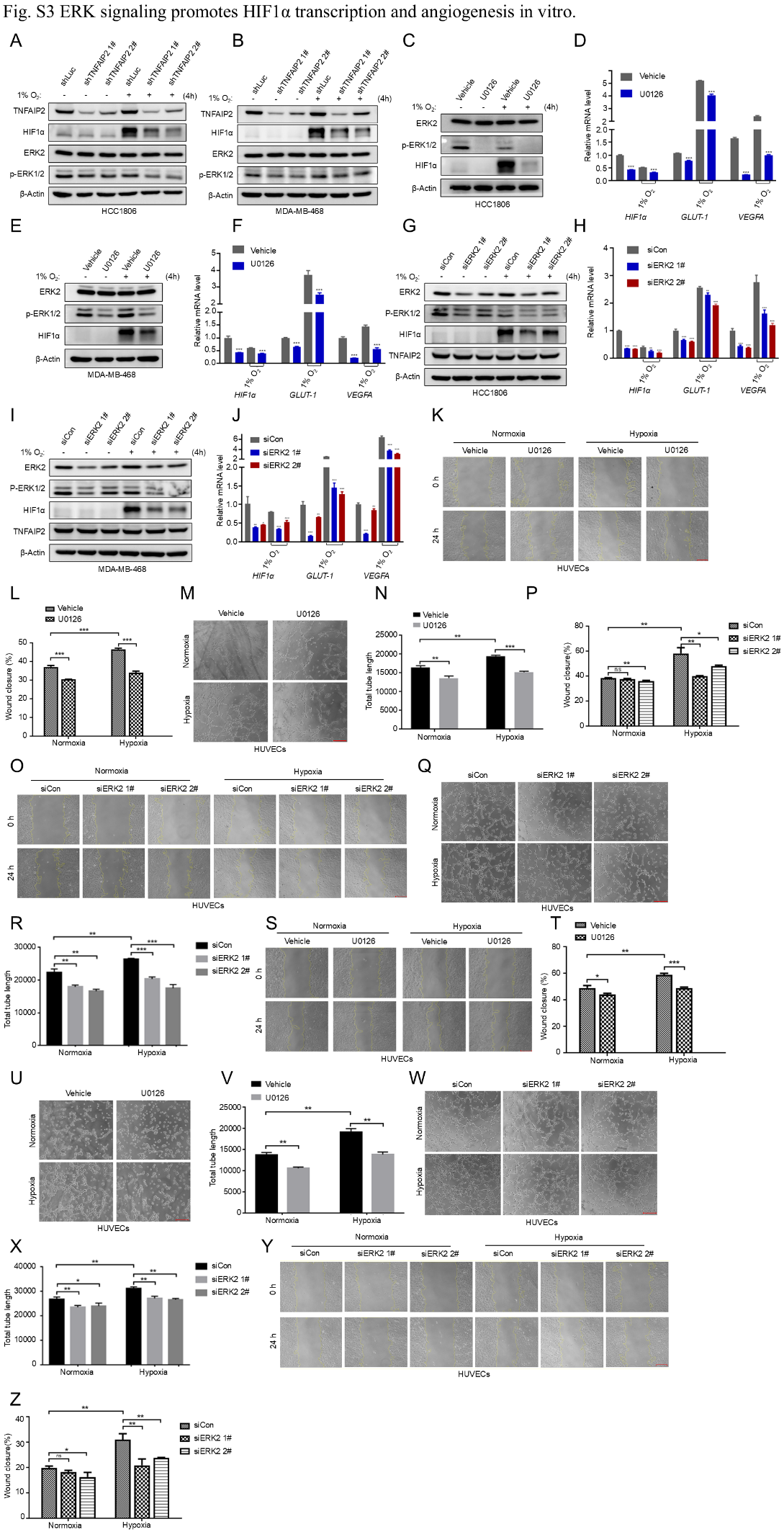


**Figure S3. ERK signaling promotes *HIF1α* transcription and angiogenesis *in vitro***

(A) Knockdown of TNFAIP2 in HCC1806 cells reduced p-ERK1/2 levels. Following stable knockdown of TNFAIP2, the cells were exposed to 1% O_2_ for 4 hours, and cell lysates were harvested for WB analysis. (B) Knockdown of TNFAIP2 in MDA-MB-468 cells reduced the p-ERK1/2 expression. (C) U0126 reduced HIF1α protein expression in HCC1806 cells. HCC1806 cells were treated with U0126 (10 μM, 12 h) and exposed to 1% O_2_ for 4 hours, and cell lysates were harvested for WB analysis. (D) U0126 reduced mRNA levels of *HIF1α*, *GLUT-1* and *VEGFA* in HCC1806 cells*.* HCC1806 cells were exposed to 1% O_2_ for 48 hours and treated with U0126 (10 μM, 12 h). Cell lysates were harvested for real-time PCR analysis. (E) U0126 reduced HIF1α protein expression in MDA-MB-468 cells. (F) U0126 reduced mRNA levels of *HIF1α,* *GLUT-1* and *VEGFA* in MDA-MB-468 cells. (G) Knockdown of ERK2 in HCC1806 cells reduced HIF1α protein expression. (H) Knockdown of ERK2 in HCC1806 cells reduced the mRNA levels of *HIF1α*, *GLUT-1* and *VEGFA*. (I) Knockdown of ERK2 in MDA-MB-468 cells reduced HIF1α protein expression. (J) Knockdown of ERK2 in MDA-MB-468 cells reduced the mRNA levels of *HIF1α*, *GLUT-1* and *VEGFA*. (K, L) The hypoxia-induced migration of HUVECs was inhibited by CM collected from HCC1806 cells treated with U0126 (10 μM, 12 h) in the wound-healing assay. Representative images are shown. (M, N) The hypoxia-induced tube formation of HUVECs was inhibited by CM collected from HCC1806 cells treated with U0126 (10 μM, 12 h). Representative images are shown. (O, P) The hypoxia-induced migration of HUVECs was inhibited by CM collected from ERK2 kncokdown HCC1806 cells in the wound-healing assay. Representative images are shown. (Q, R) The hypoxia-induced tube formation of HUVECs was inhibited by CM collected from ERK2 kncokdown HCC1806 cells. Representative images are shown. (S, T) The hypoxia-induced migration of HUVECs was inhibited by CM collected from MDA-MB-468 cells treated with U0126 (10 μM, 12 h) in the wound-healing assay. Representative images are shown. (U, V) The hypoxia-induced tube formation of HUVECs was inhibited by CM collected from MDA-MB-468 cells treated with U0126 (10 μM, 12 h). Representative images are shown. (W, X) The hypoxia-induced migration of HUVECs was inhibited by CM collected from ERK2 kncokdown MDA-MB-468 cells in the wound-healing assay. Representative images are shown. (Y, Z) The hypoxia-induced tube formation of HUVECs was inhibited by CM collected from ERK2 kncokdown MDA-MB-468 cells. Representative images are shown. Scale bar, 200 μm.* *P* < 0.05, ** *P* < 0.01 and *** *P* < 0.001; ns, not significant.


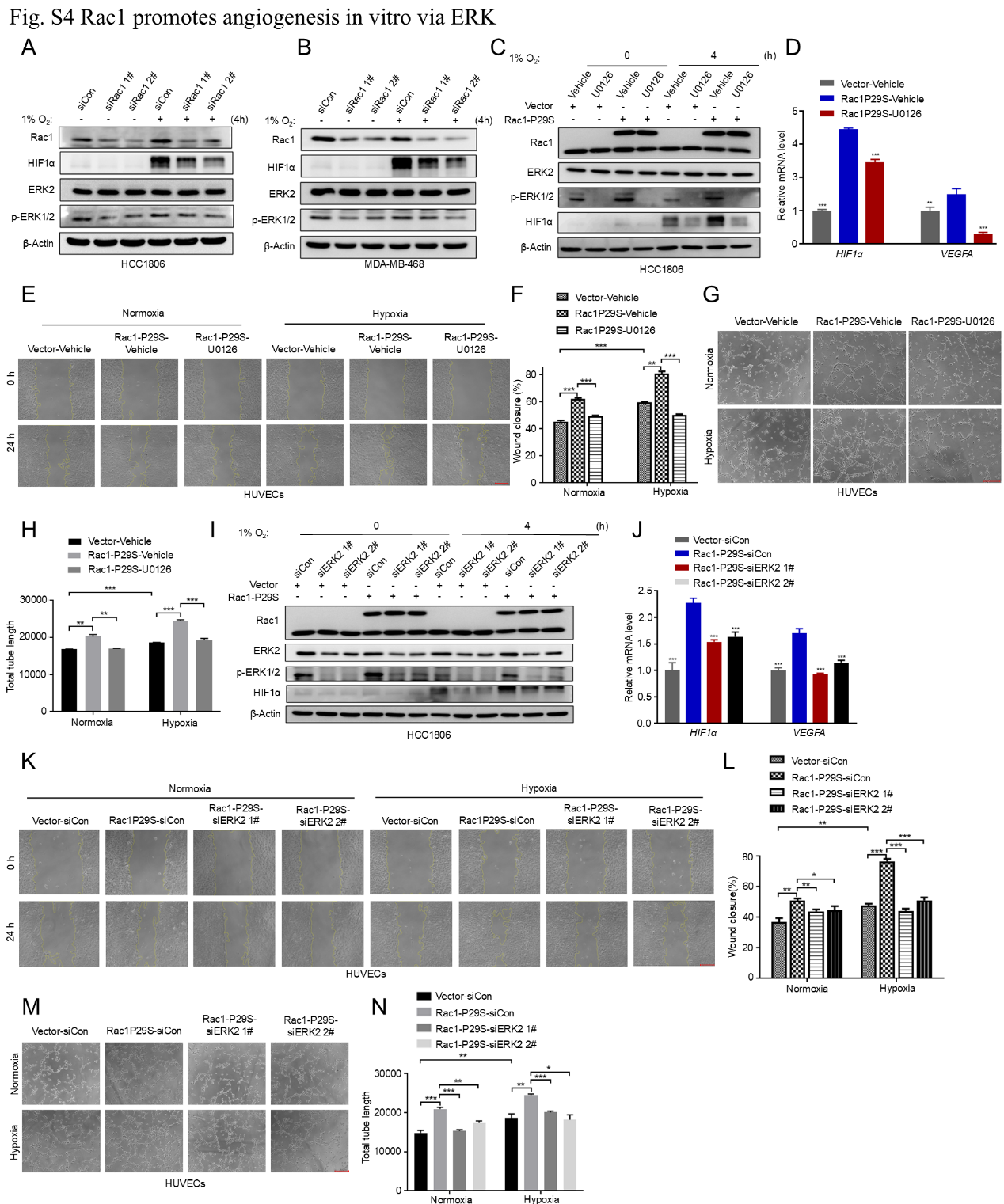
 **Figure S4. Rac1 promotes angiogenesis *in vitro* via ERK**

(A) Knockdown of Rac1 in HCC1806 cells reduced the p-ERK1/2 expression. (B) Knockdown of Rac1 in MDA-MB-468 cells reduced the p-ERK1/2 expression level. (C) U0126 abolished Rac1-P29S-induced HIF1α upregulation. HCC1806 cells with stable Rac1-P29S overexpression were treated U0126 (10 μM, 12 h) and exposed to 1% O_2_ for 4 hours. Cell lysates were harvested for WB analysis. (D) U0126 abolished Rac1-P29S-induced *HIF1α* and *VEGFA* upregulation. HCC1806 cells with stable Rac1-P29S overexpression were treated U0126 (10 μM, 12 h), and cell lysates were harvested for real-time PCR analysis. (E, F) U0126 impeded the Rac1-P29S overexpression induced migration of HUVECs in the wound-healing assay. Representative images are shown. (G, H) U0126 impeded the Rac1-P29S overexpression induced tube formation of HUVECs. Representative images are shown. (I) ERK2 knockdown abolished Rac1-P29S-induced HIF1α and p-ERK1/2 upregulation. (J) ERK2 knockdown abolished Rac1-P29S-induced *HIF1α* and *VEGFA* mRNA upregulation. (K, L) ERK2 knockdown impeded the Rac1-P29S overexpression induced migration of HUVECs in the wound-healing assay. Representative images are shown. (M, N) ERK2 knockdown impeded the Rac1-P29S overexpression indcued tube formation of HUVECs. Representative images are shown. Scale bar, 200 μm.* *P* < 0.05, ** *P* < 0.01 and *** *P* < 0.001; ns, not significant.


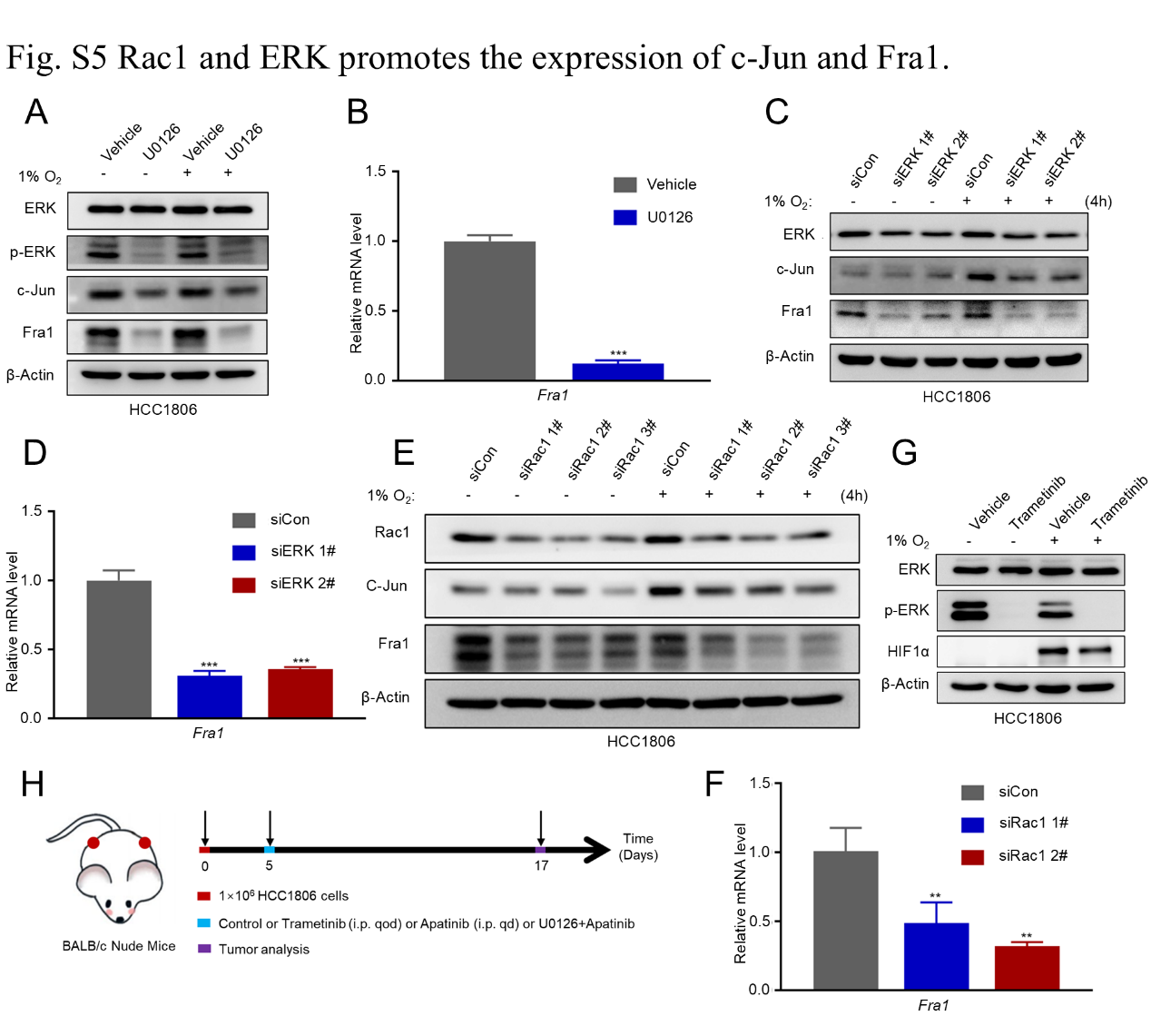
 **Figure S5. Rac1 and ERK promotes the expression of c-Jun and Fra1**

(A) U0126 reduced c-Jun and Fra1 protein expression in HCC1806 cells. HCC1806 cells were treated with U0126 (10 μM, 12 h) and exposed to 1% O_2_ for 4 hours, cell lysates were harvested for WB analysis. (B) U0126 reduced mRNA levels of *Fra1* in HCC1806 cells. HCC1806 cells were exposed to 1% O_2_ for 48 hours and treated with U0126 (10 μM, 12 h). Cell lysates were harvested for real-time PCR analysis. (C) Knockdown of ERK2 in HCC1806 cells reduced c-Jun and Fra1 protein expression. (D) Knockdown of ERK2 in HCC1806 cells reduced the mRNA levels of *Fra1*. (E) Knockdown of Rac1 in HCC1806 cells reduced c-Jun and Fra1 protein expression. (F) Knockdown of Rac1 in HCC1806 cells reduced the mRNA levels of *Fra1*. (G) Trametinib reduced HIF1α protein expression in HCC1806 cells. HCC1806 cells were treated with trametinib (100 nM, 24 h) and exposed to 1% O_2_ for 24 hours, cell lysates were harvested for WB analysis. (H) Schematic diagram of orthotopic tumor model construction in nude mice and administration time and frequency.

**Supplementary Table 1. Sequences of shRNAs and siRNAs**

| TNFAIP2-shRNA#1 | 5’-GACUUGGGCUCACAGAUAA-3’ |
| --- | --- |
| TNFAIP2-shRNA#2 | 5’-GAUUGAGGUGGCCACUUAU-3’ |
| Fra1-shRNA#1 | 5’-GTCGAAGGCCTTGTGAACA-3’ |
| Fra1-shRNA#2 | 5’-GCTCATCGCAAGAGTAGCA-3’ |
| Luciferase-shRNA | 5’-CUUACGCUGAGUACUUCGA-3’ |
| Rac1-siRNA#1 | 5’-GCAAAGTGGTATCCTGAGG-3’ |
| Rac1-siRNA#2 | 5’-GAGGAAGAGAAAATGCCTG-3’ |
| Rac1-siRNA#3 | 5’-CGGCACCACUGUCCCAACA-3’ |
| ERK2-siRNA#1 | 5’-GAACATCATTGGAATCAAT-3’ |
| ERK2-siRNA#2 | 5’-GCTACACCAACCTCTCGTA-3’ |
| HIF1α-siRNA#1 | 5’-AAGAGGTGGATATGTCTGG-3’ |
| HIF1α-siRNA#2 | 5’-CGTCGAAAAGAAAAGTCTCTT-3’ |
| c-Jun-siRNA#1 | 5’-CCAAGAACGTGACAGATGA-3’ |
| c-Jun-siRNA#2 | 5’-CGCAGCAGTTGCAAACATT-3’ |

**Supplementary Table 2. Sequences of Primers for qRT-PCR**

| **Name** | **Forward(5’-3’)** | **Reverse(5’-3’)** |
| --- | --- | --- |
| 18S rRNA | CTCAACACGGGAAACCTCAC | CGCTCCACCAACTAAGAACG |
| TNFAIP2 | TTTCCGGGAGGAGCTCATGG | CAGGTGGCCTTTGCTGAAGT |
| Rac1 | CCCTATCCTATCCGCAAACAGATG | CAAATGATGCAGGACTCACAAGGG |
| ERK2 | CAACACCTCAGCAATGACCA | GATCACAGGTGGTGTTGAGC |
| Fra1 | CAGGCGGAGACTGACAAACTG | TCCTTCCGGGATTTTGCAGAT |
| HIF1α | AAGTCTGCAACATGGAAGGTAT | TGAGGAATGGGTTCACAAATC |
| GLUT-1 | TCGTCGGCATCCTCATCGCC | CCGGTTCTCCTCGTTGCGGT |
| VEGFA | AAGGAGGAGGGCAGAATCAT | ATCTGCATGGTGATGTTGGA |

**Supplementary Table 3. Sequences of Primers used for amplifying the region of *HIF1α* gene promoter**

| **Name** | **Forward(5’-3’)** | **Reverse(5’-3’)** |
| --- | --- | --- |
| -432 to -422 | AGGCCGAGGAGAAAGAGAGCA | ACTCTGCGCCTTCTCCGAA |
| -20 to -10 | TGACGCTGCCTCAGCTCCTCA | CTCTCCTCAGGTGGCTTGTCA |
