# Supplementary material for: TNFAIP2 promotes HIF1α transcription and breast cancer angiogenesis by activating the Rac1-ERK-AP1 signaling axis
Source: Cell Death Dis. 2024 Nov 13;15(11):821. doi: 10.1038/s41419-024-07223-2 (PMC11557851; doi:10.1038/s41419-024-07223-2)
Supplement: Supplementary file 2 — Original western blots [file 41419_2024_7223_MOESM2_ESM.zip › Figure 5.pptx]

## Slide 1
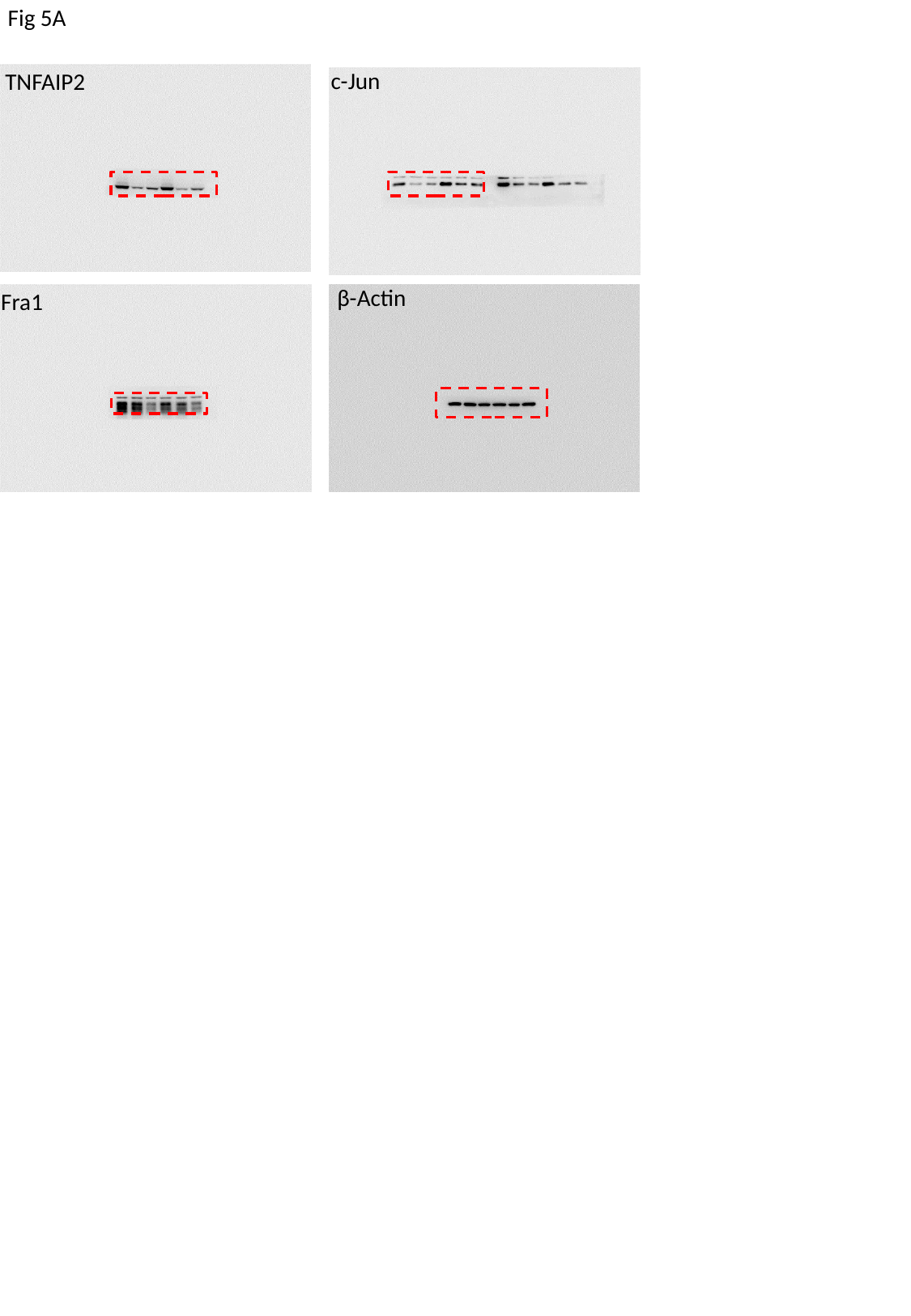

Fig 5A
c-Jun
TNFAIP2
β-Actin
Fra1

## Slide 2
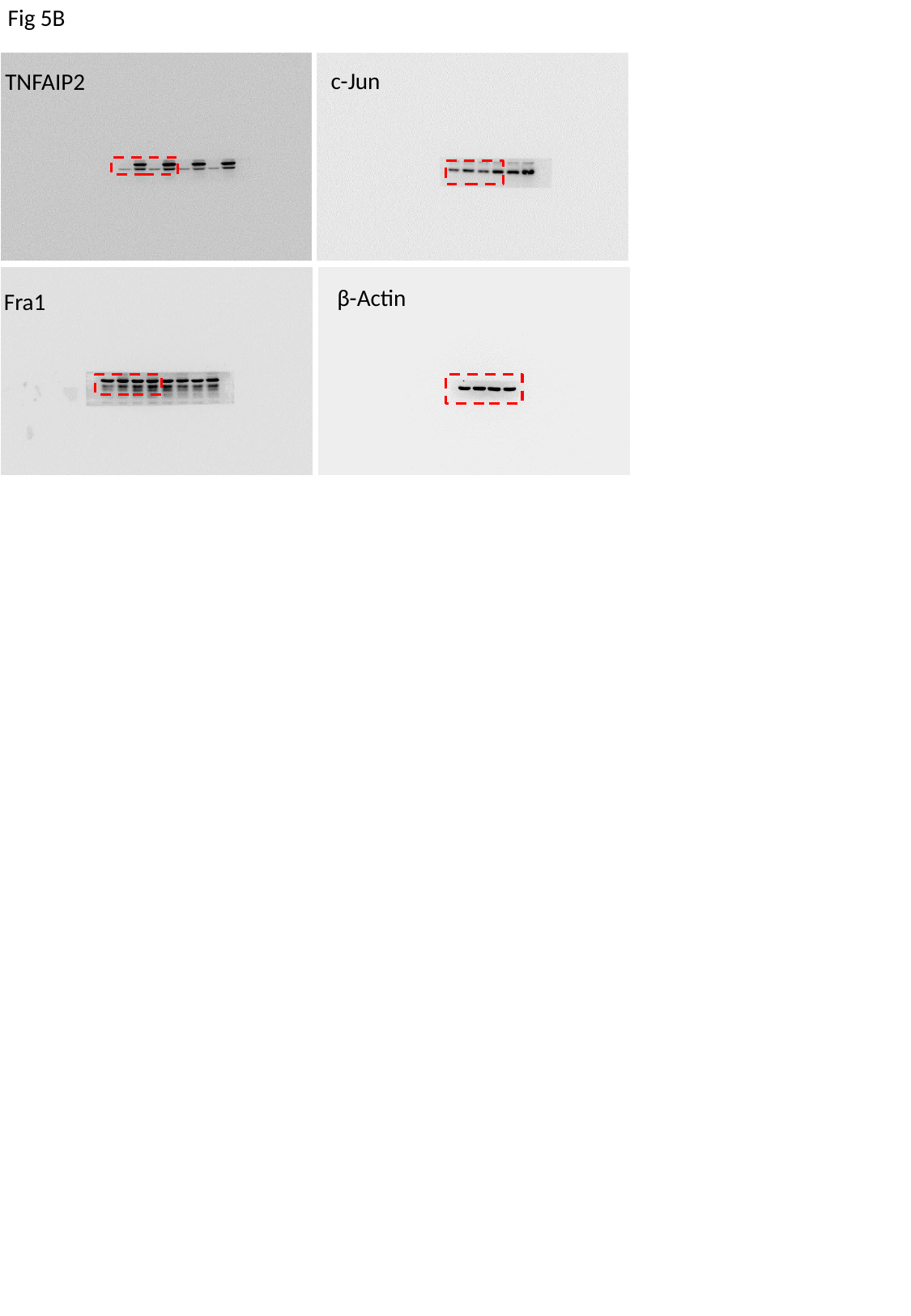

Fig 5B
c-Jun
TNFAIP2
β-Actin
Fra1

## Slide 3
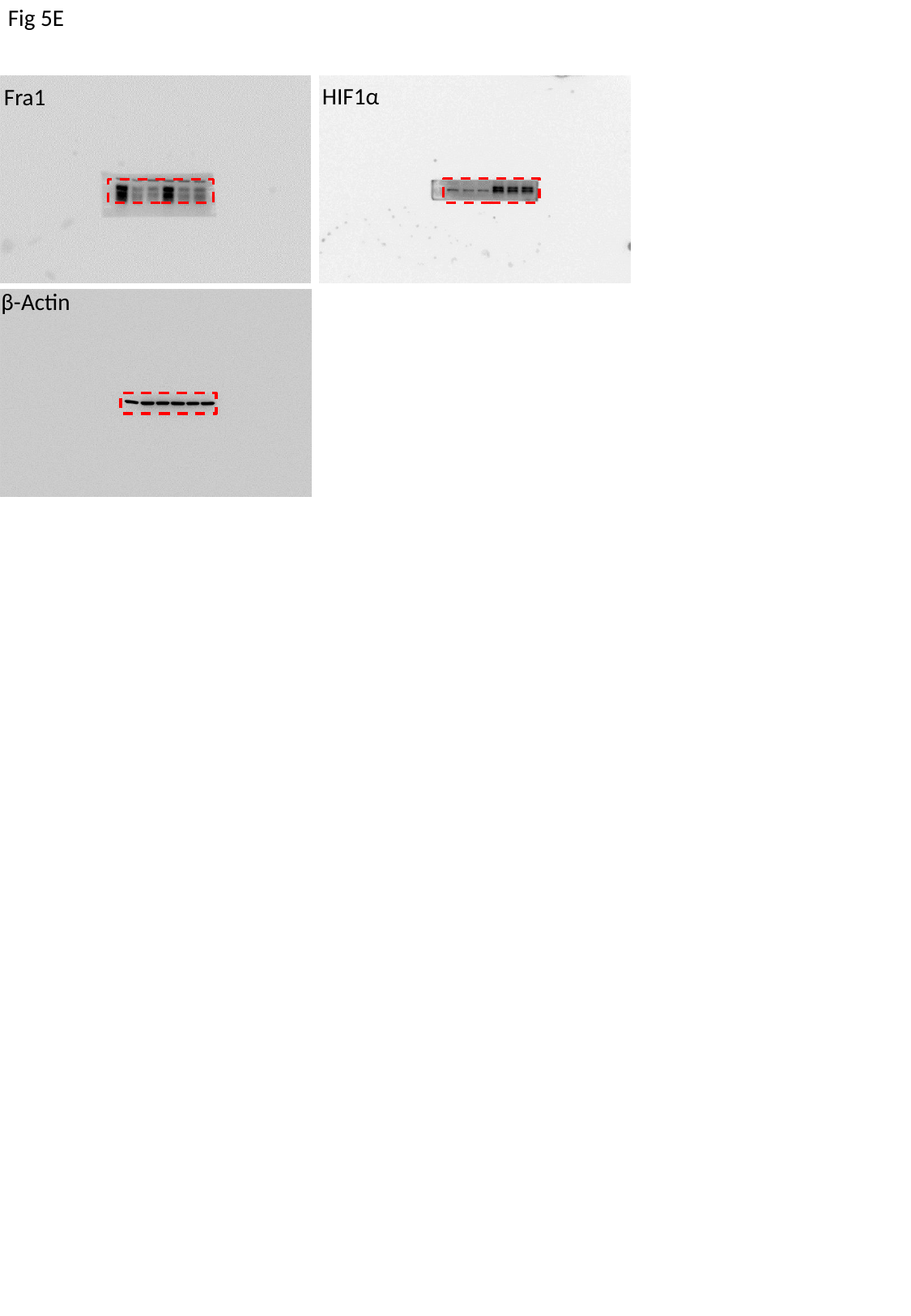

Fig 5E
HIF1α
Fra1
β-Actin

## Slide 4
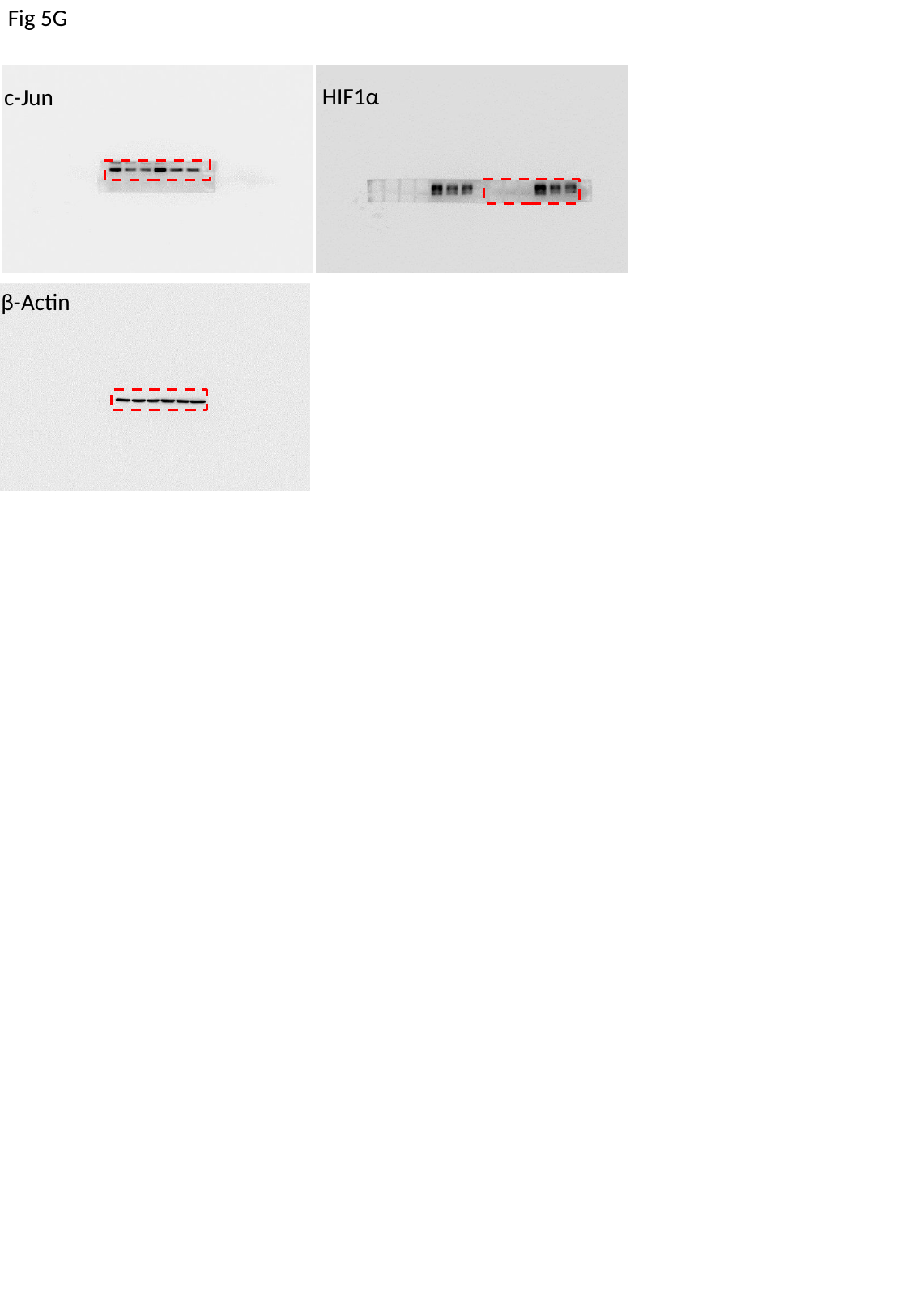

Fig 5G
HIF1α
c-Jun
β-Actin

## Slide 5
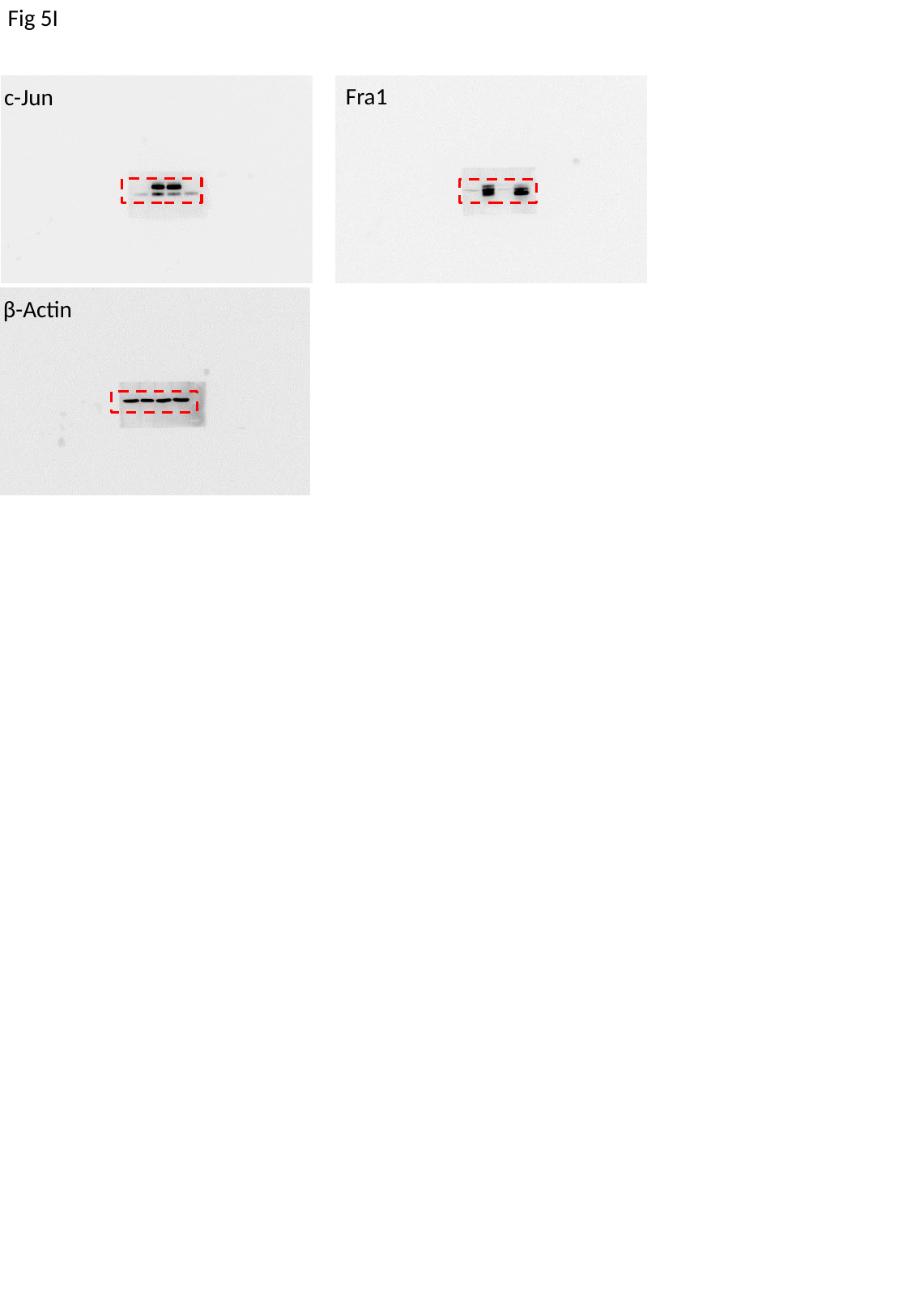

Fig 5I
Fra1
c-Jun
β-Actin

## Slide 6
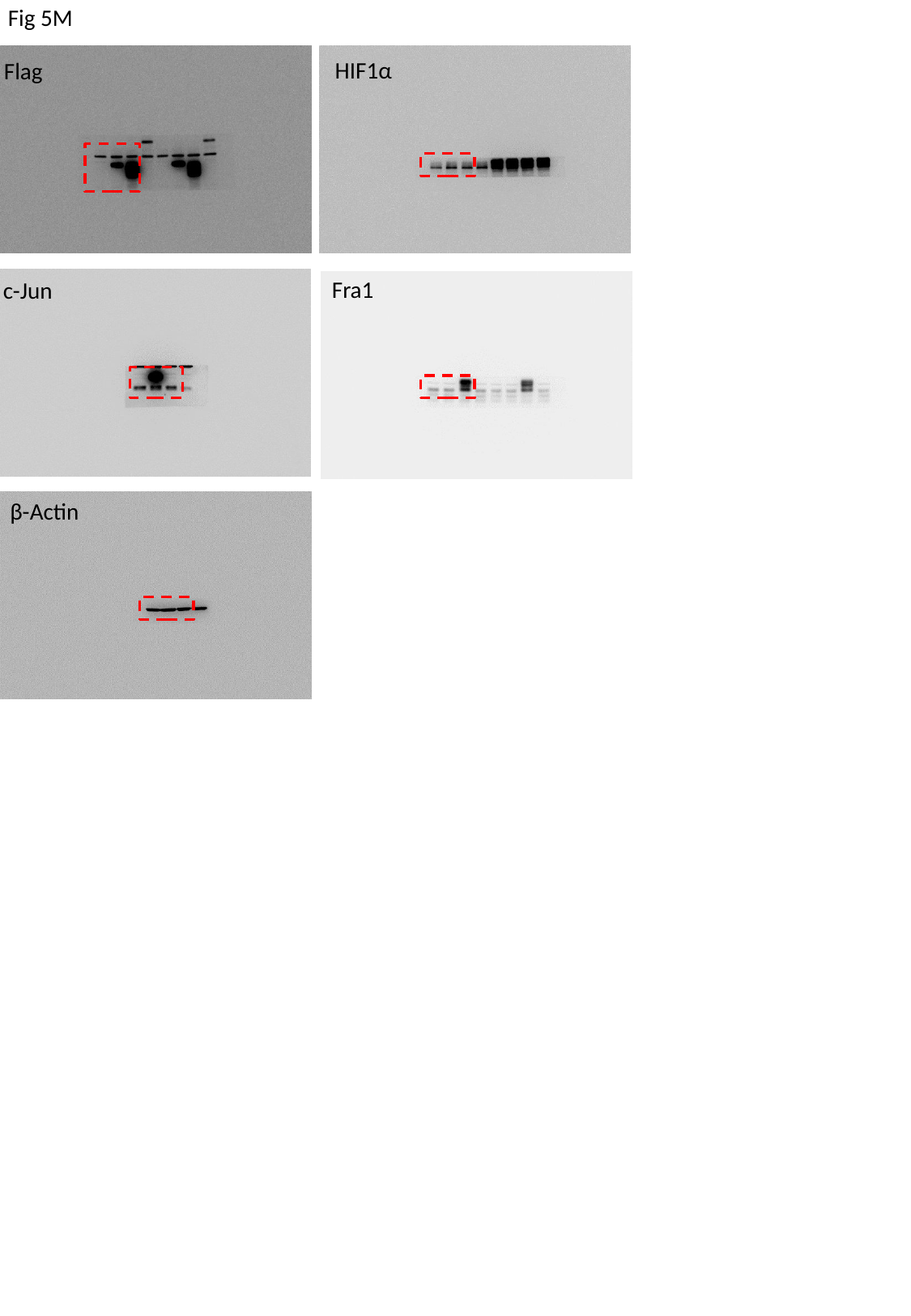

Fig 5M
HIF1α
Flag
Fra1
c-Jun
β-Actin

## Slide 7
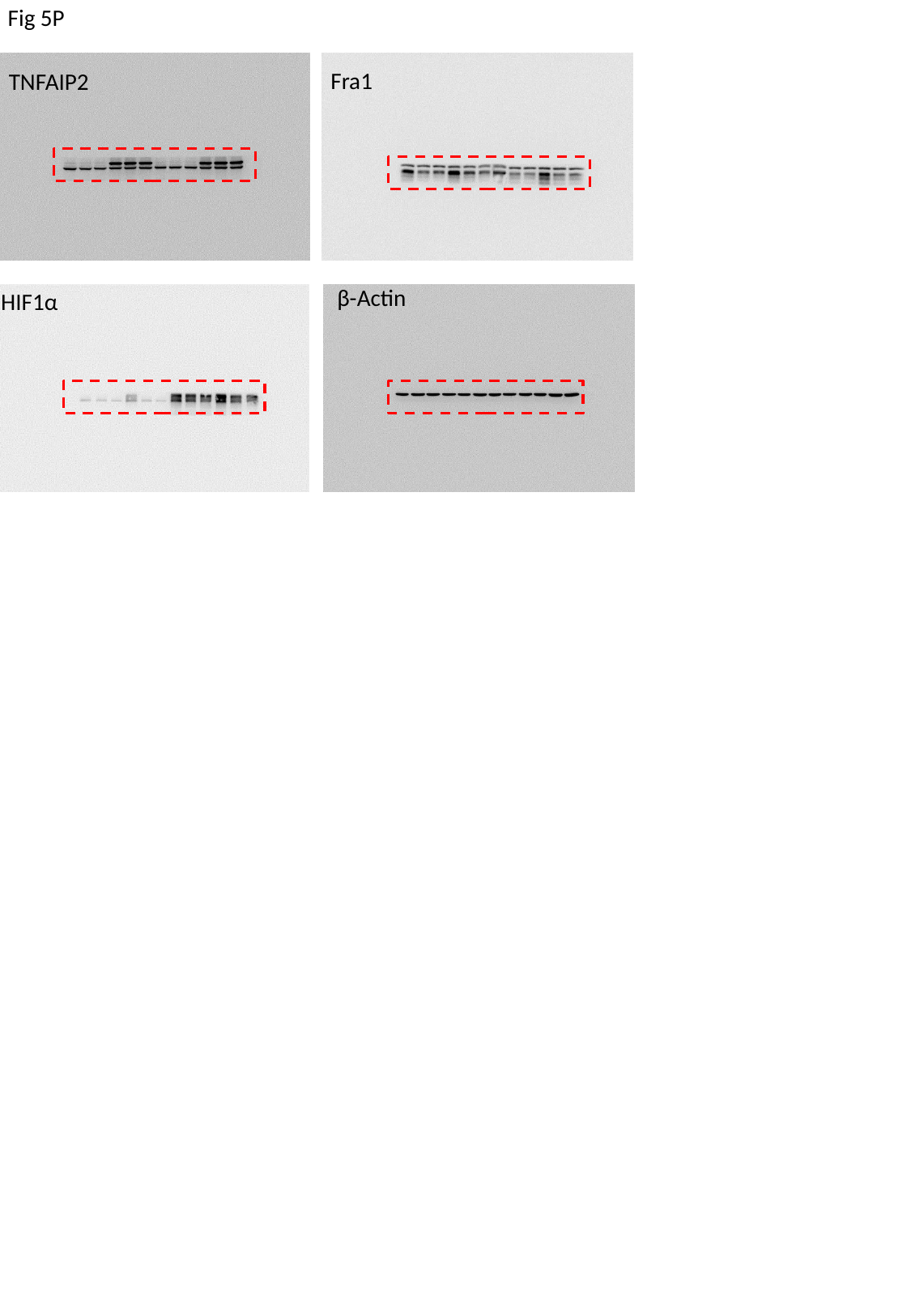

Fig 5P
Fra1
TNFAIP2
β-Actin
HIF1α
