# Supplementary material for: TNFAIP2 promotes HIF1α transcription and breast cancer angiogenesis by activating the Rac1-ERK-AP1 signaling axis
Source: Cell Death Dis. 2024 Nov 13;15(11):821. doi: 10.1038/s41419-024-07223-2 (PMC11557851; doi:10.1038/s41419-024-07223-2)
Supplement: Supplementary file 2 — Original western blots [file 41419_2024_7223_MOESM2_ESM.zip › Figure S3.pptx]

## Slide 1
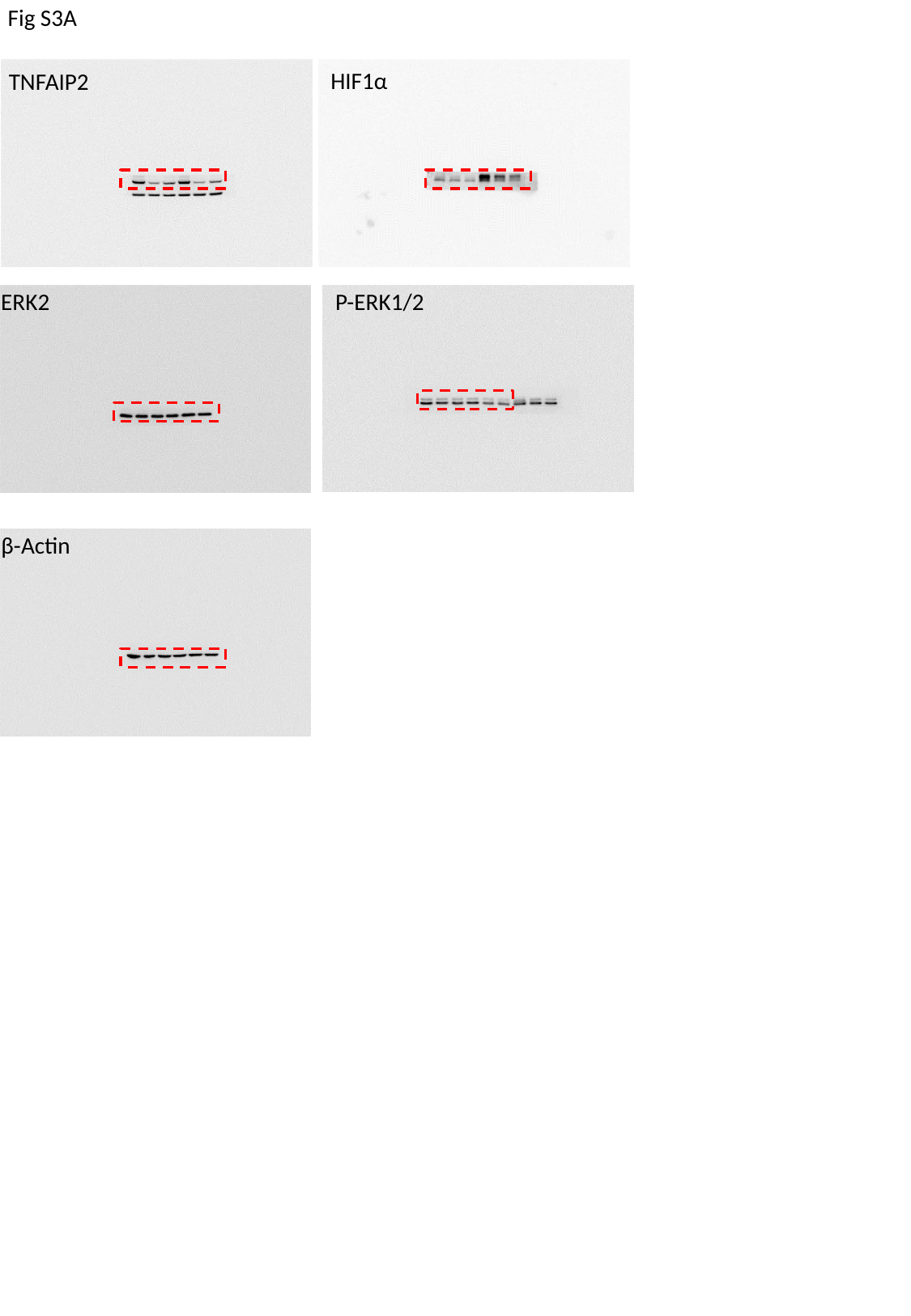

Fig S3A
HIF1α
TNFAIP2
P-ERK1/2
ERK2
β-Actin

## Slide 2
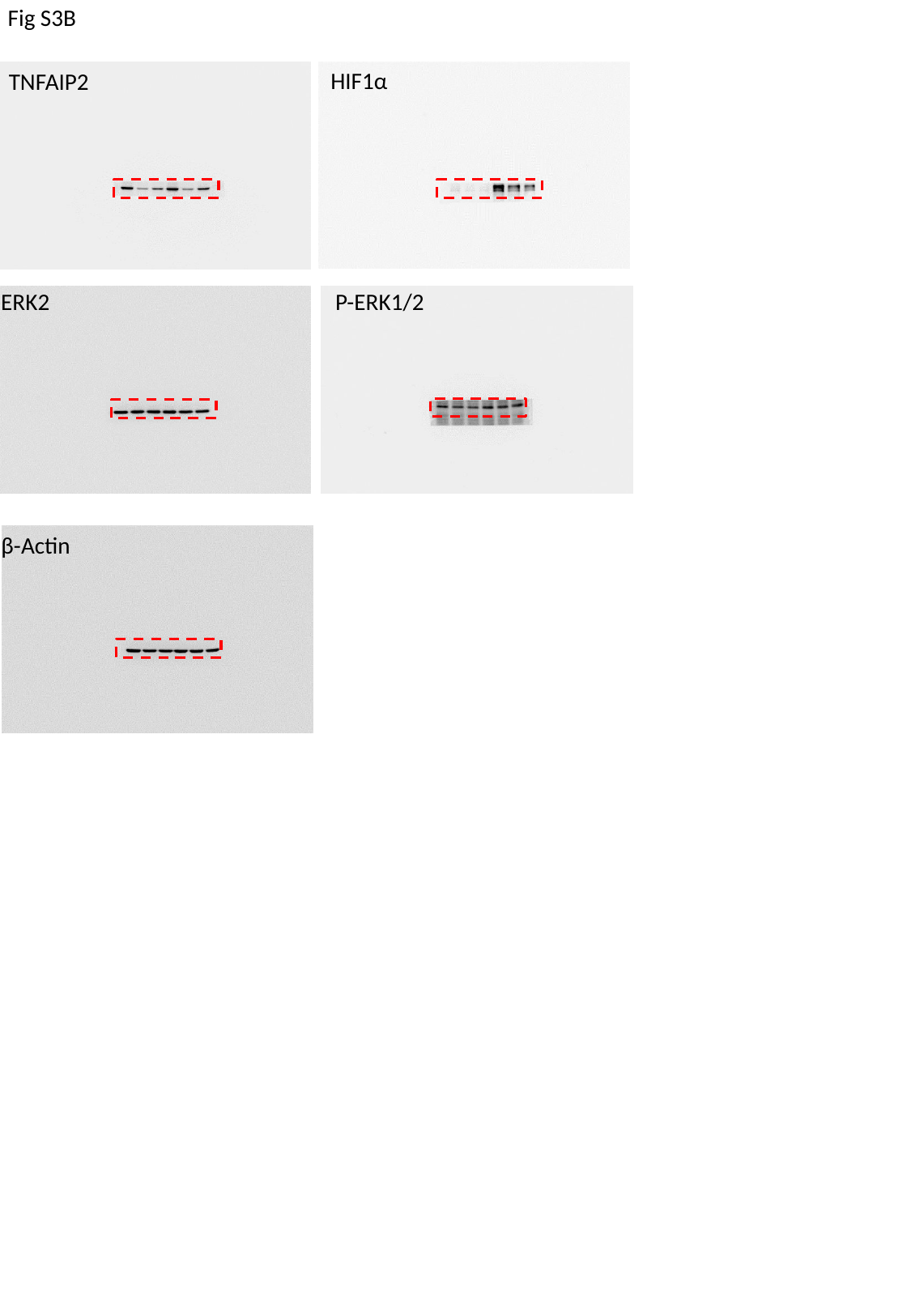

Fig S3B
HIF1α
TNFAIP2
P-ERK1/2
ERK2
β-Actin

## Slide 3
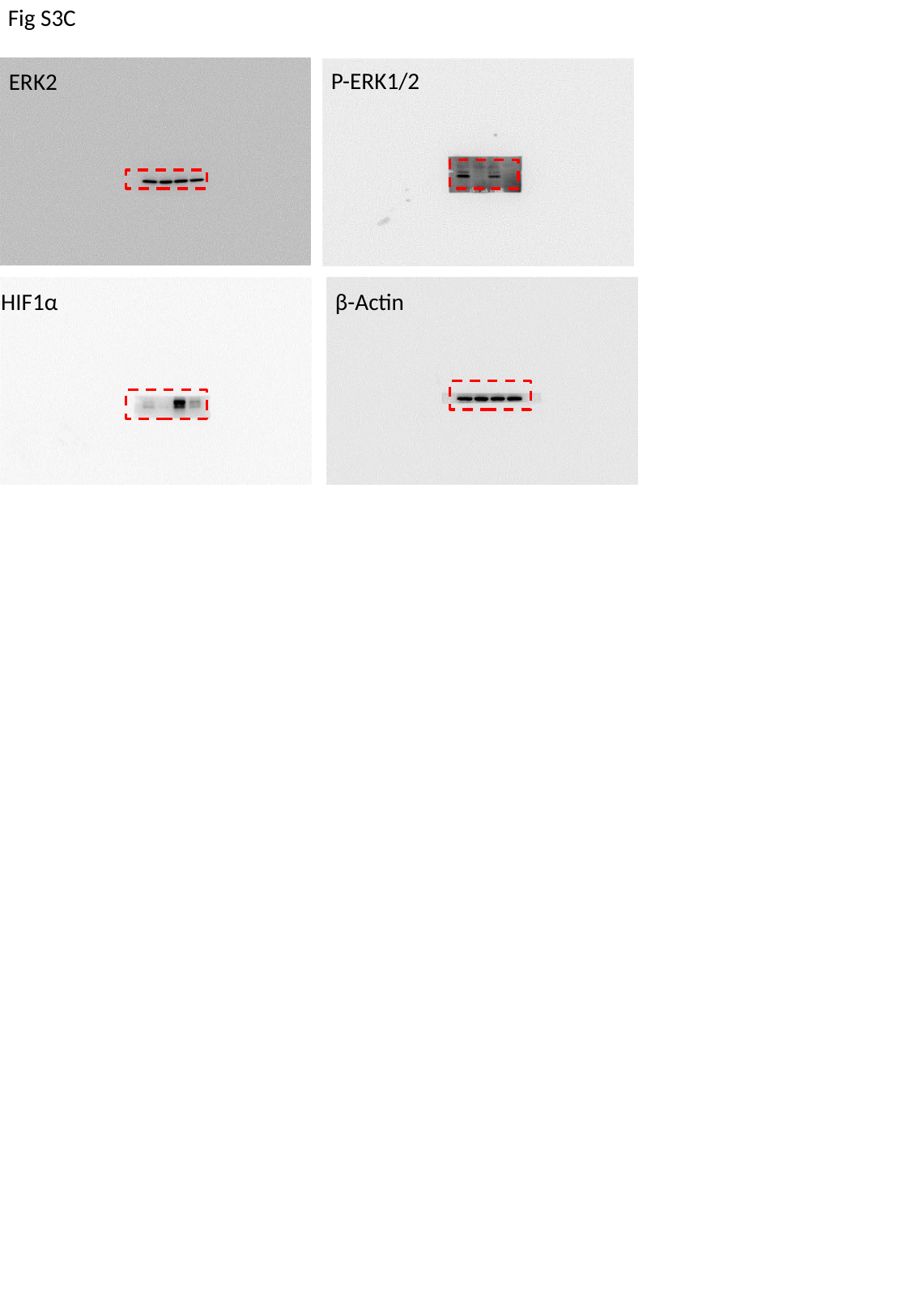

Fig S3C
P-ERK1/2
ERK2
β-Actin
HIF1α

## Slide 4
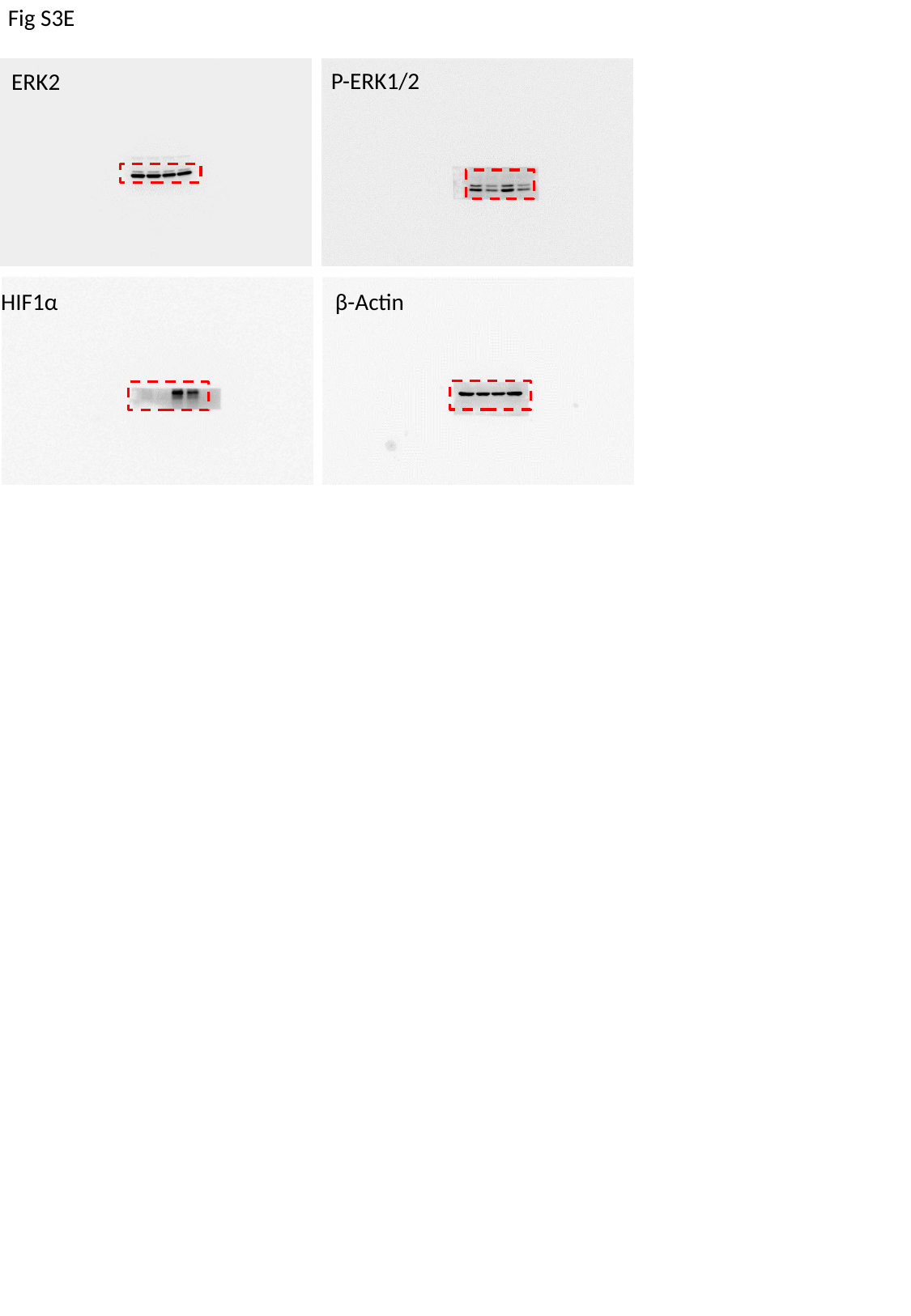

Fig S3E
P-ERK1/2
ERK2
β-Actin
HIF1α

## Slide 5
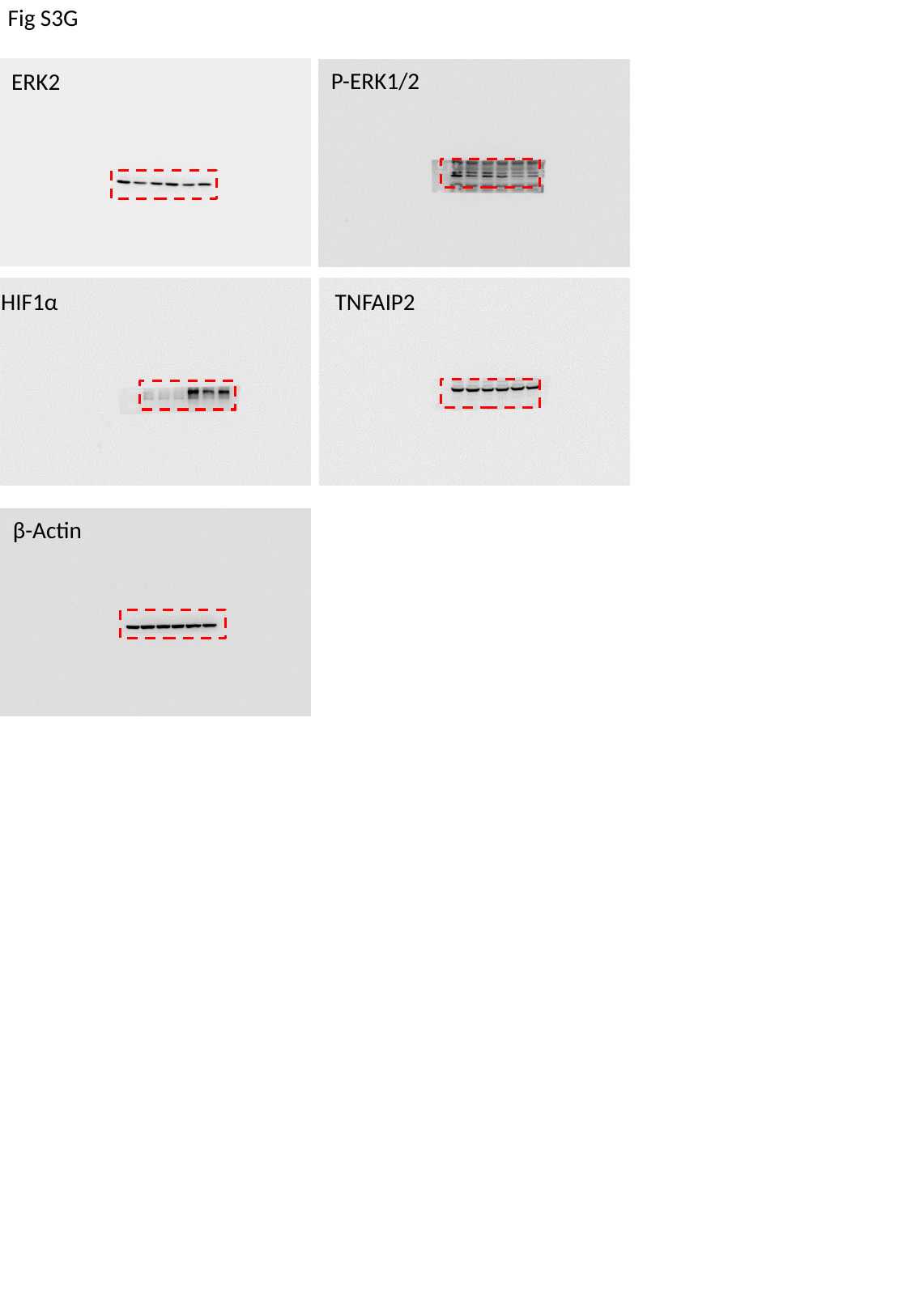

Fig S3G
P-ERK1/2
ERK2
TNFAIP2
HIF1α
β-Actin

## Slide 6
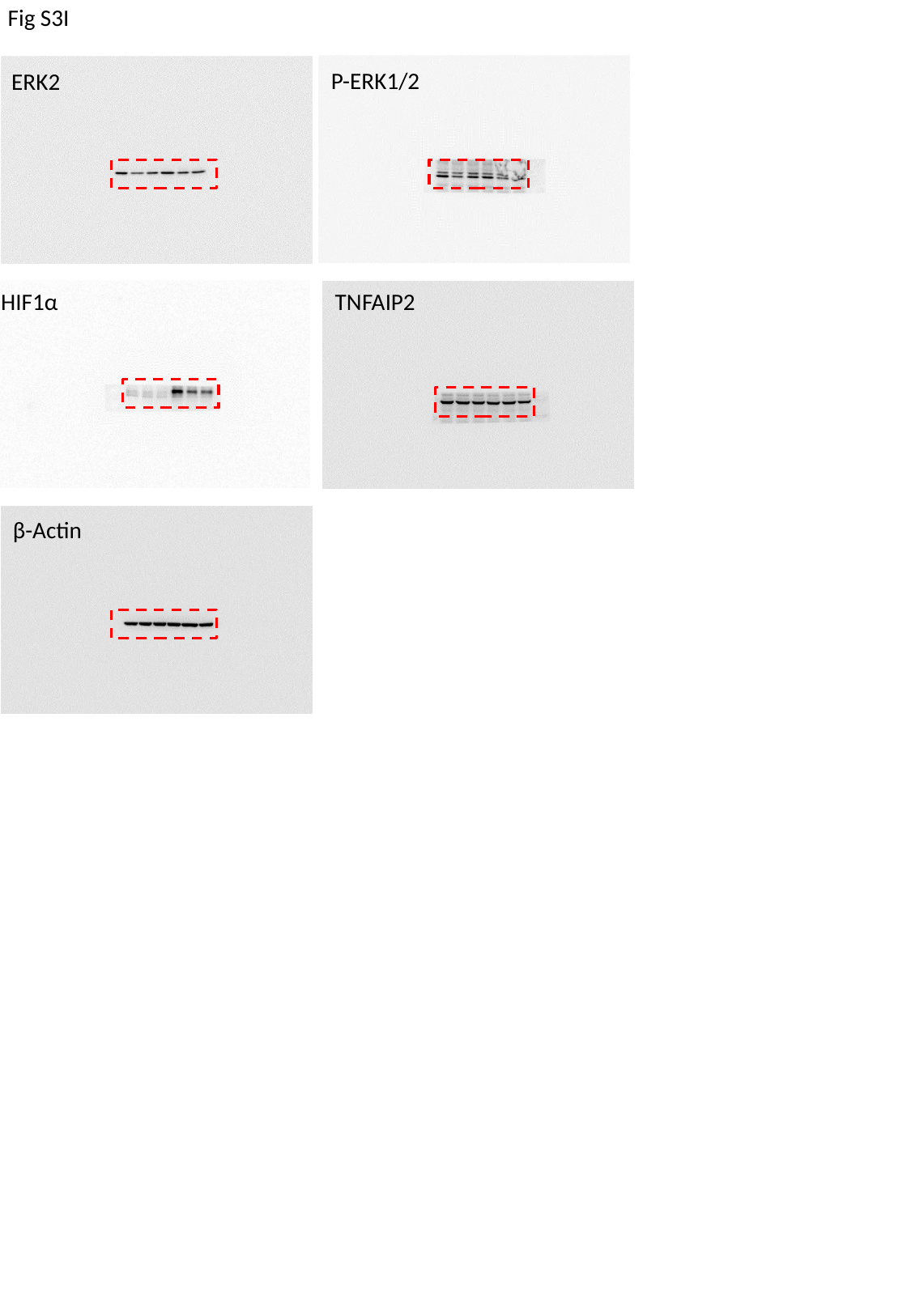

Fig S3I
P-ERK1/2
ERK2
TNFAIP2
HIF1α
β-Actin
