# Supplementary material for: TNFAIP2 promotes HIF1α transcription and breast cancer angiogenesis by activating the Rac1-ERK-AP1 signaling axis
Source: Cell Death Dis. 2024 Nov 13;15(11):821. doi: 10.1038/s41419-024-07223-2 (PMC11557851; doi:10.1038/s41419-024-07223-2)
Supplement: Supplementary file 2 — Original western blots [file 41419_2024_7223_MOESM2_ESM.zip › Figure S4.pptx]

## Slide 1
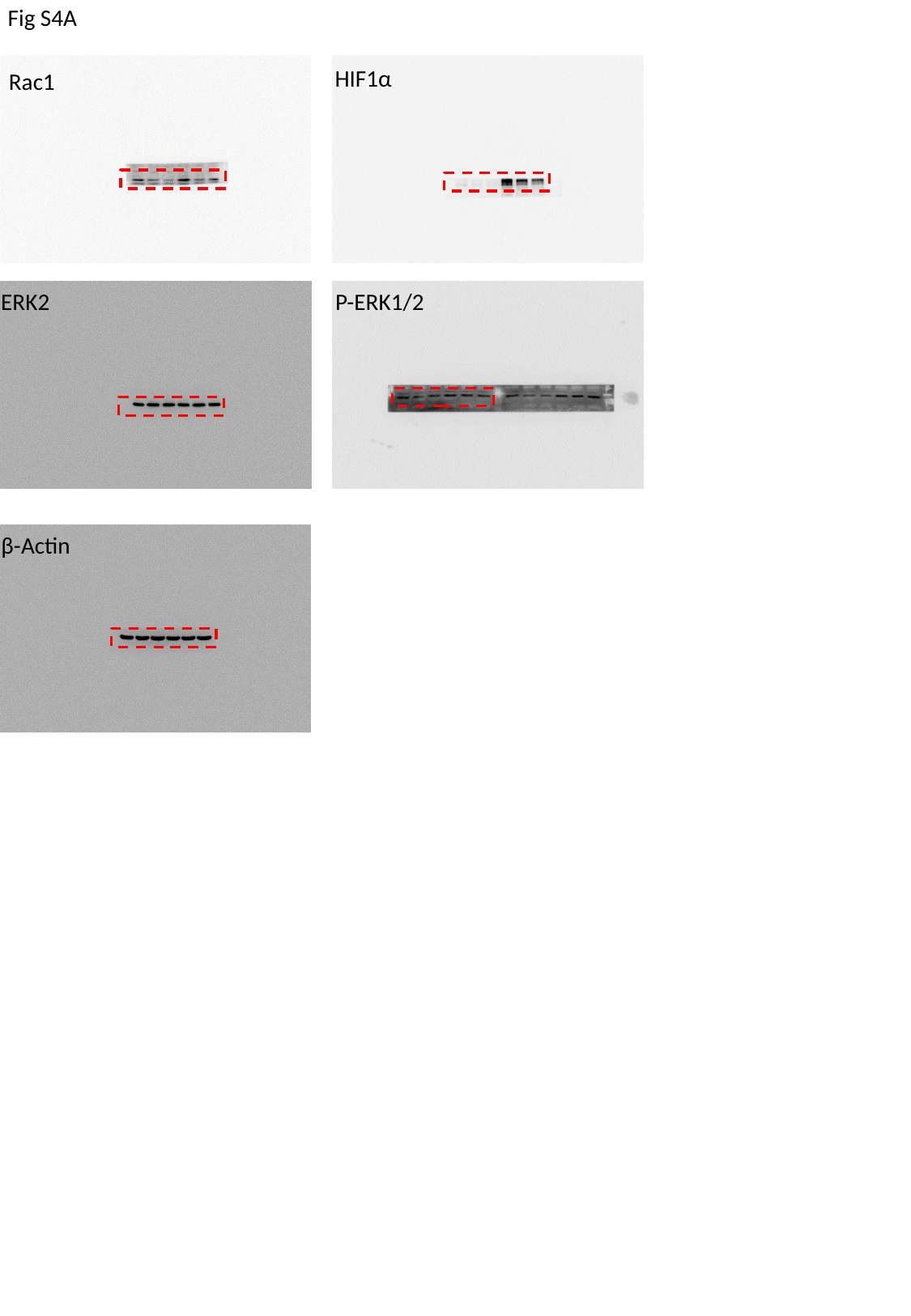

Fig S4A
HIF1α
Rac1
P-ERK1/2
ERK2
β-Actin

## Slide 2
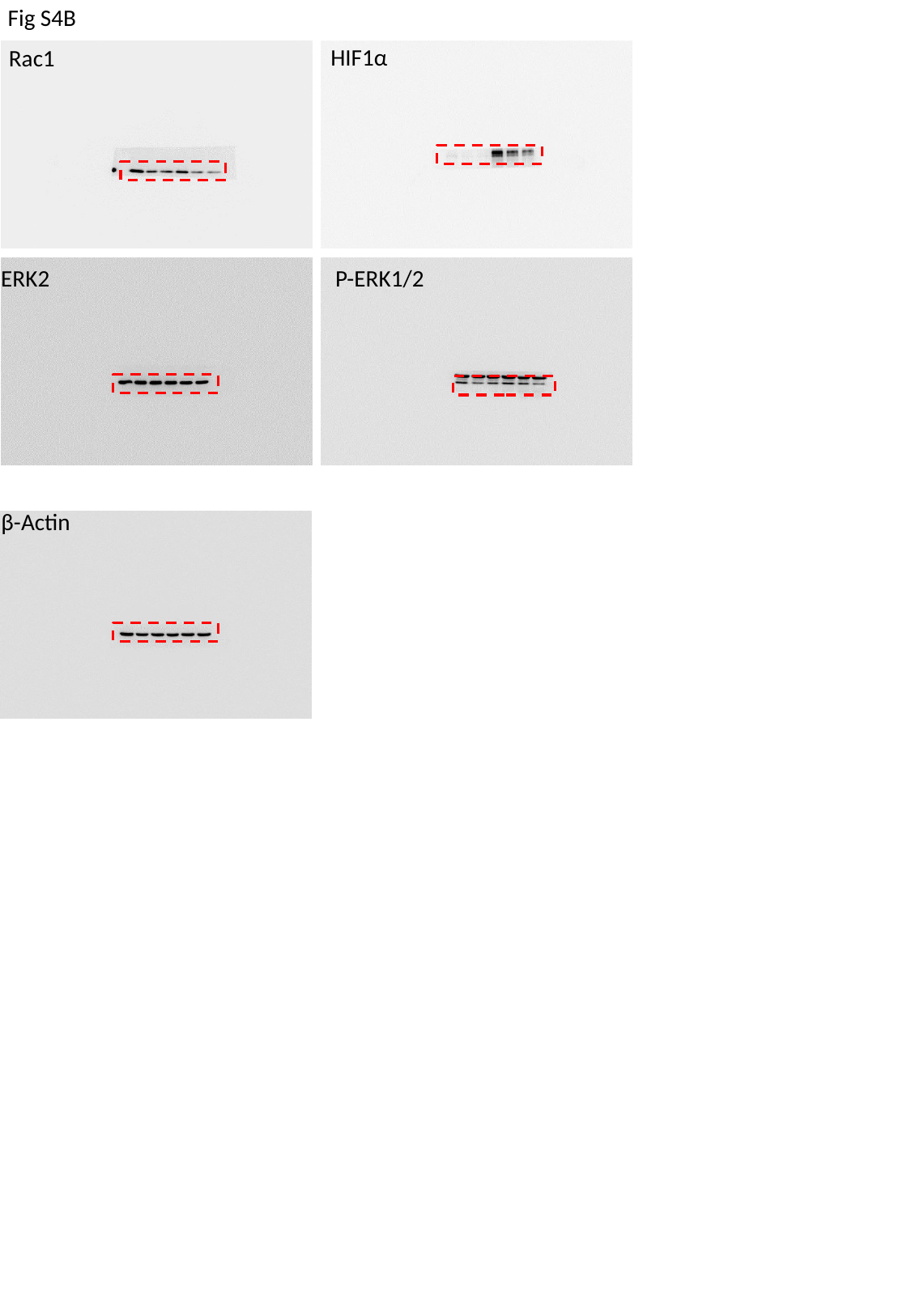

Fig S4B
HIF1α
Rac1
P-ERK1/2
ERK2
β-Actin

## Slide 3
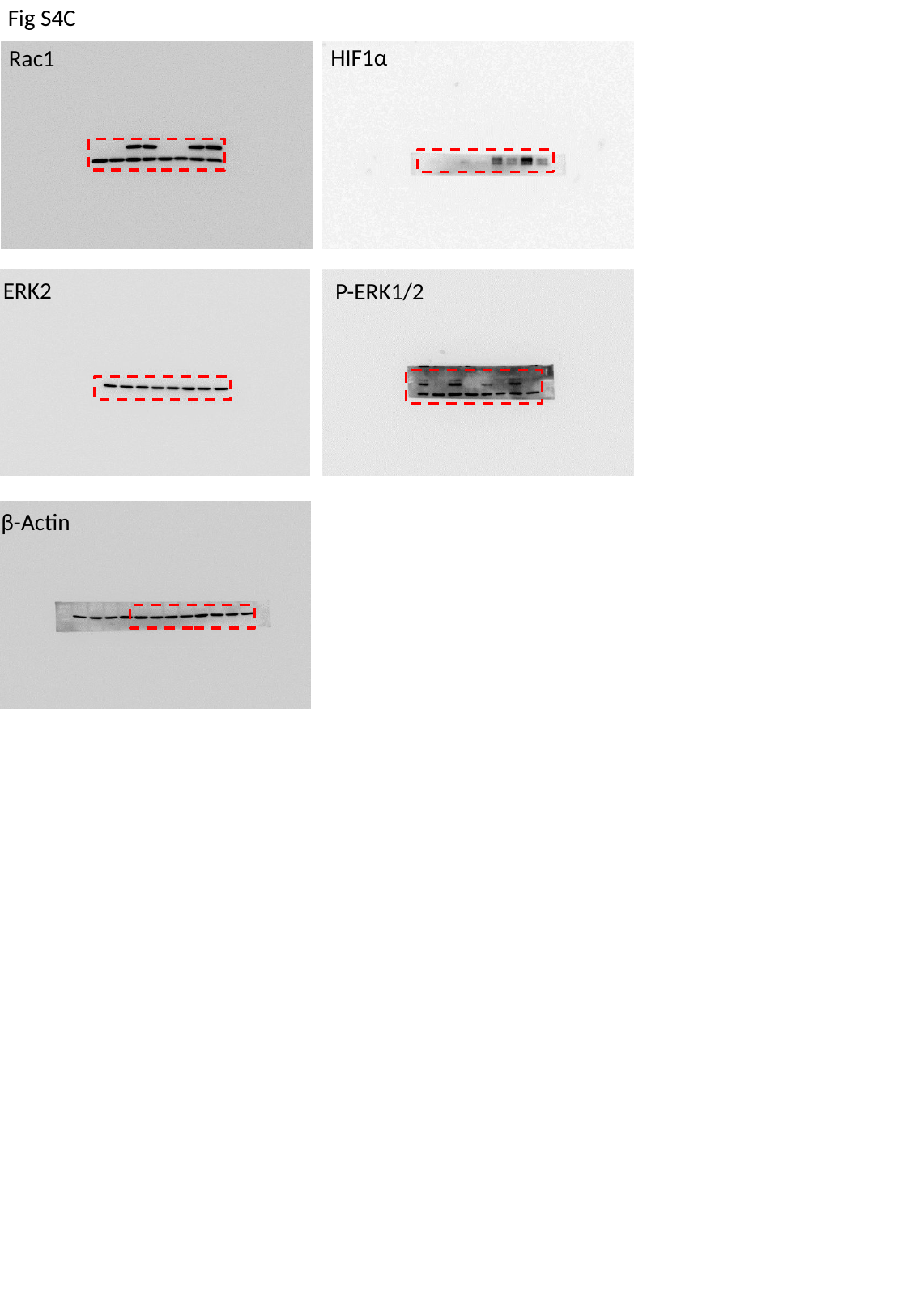

Fig S4C
HIF1α
Rac1
ERK2
P-ERK1/2
β-Actin

## Slide 4
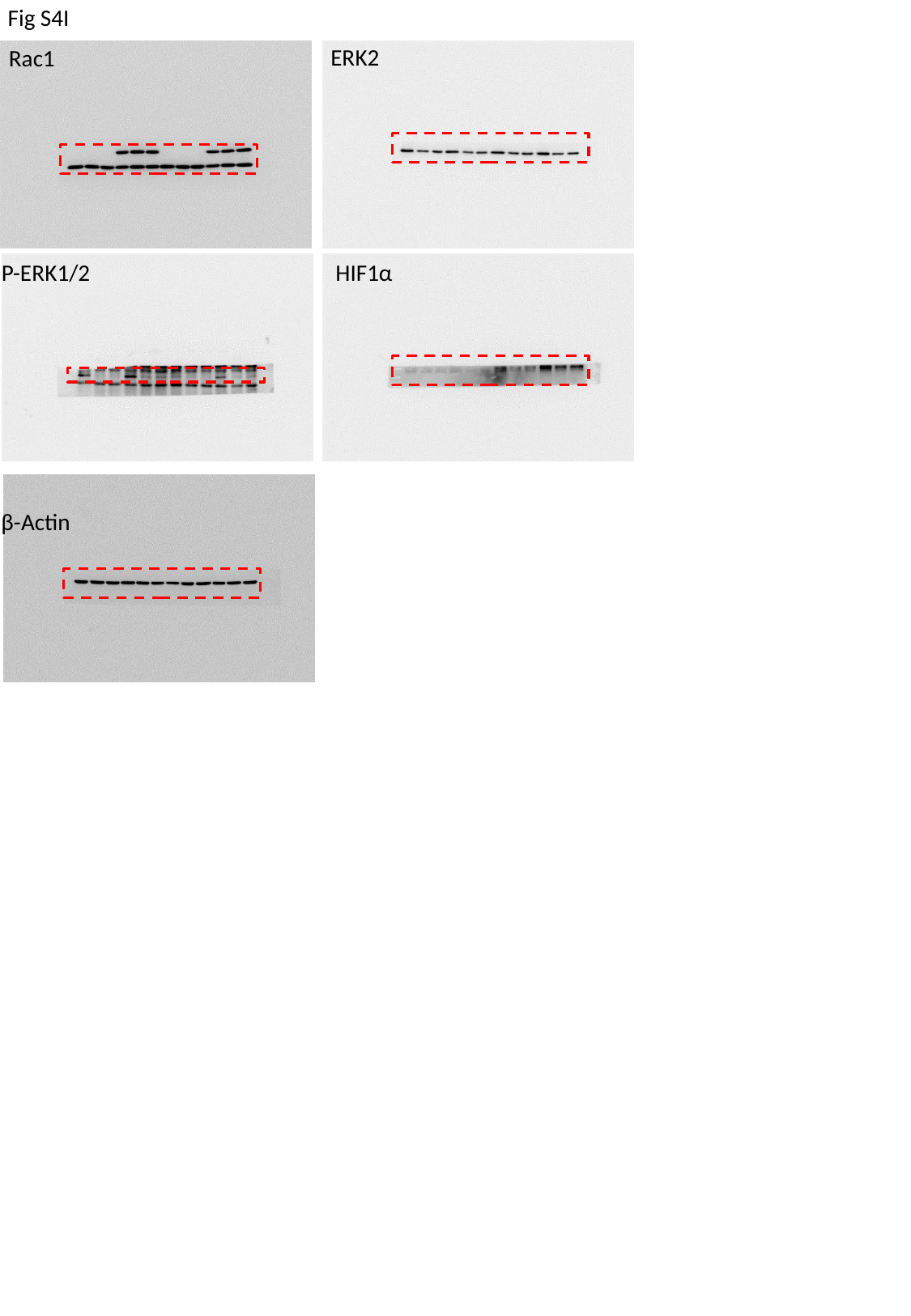

Fig S4I
ERK2
Rac1
HIF1α
P-ERK1/2
β-Actin
