# Supplementary material for: TNFAIP2 promotes HIF1α transcription and breast cancer angiogenesis by activating the Rac1-ERK-AP1 signaling axis
Source: Cell Death Dis. 2024 Nov 13;15(11):821. doi: 10.1038/s41419-024-07223-2 (PMC11557851; doi:10.1038/s41419-024-07223-2)
Supplement: Supplementary file 2 — Original western blots [file 41419_2024_7223_MOESM2_ESM.zip › Figure S5.pptx]

## Slide 1
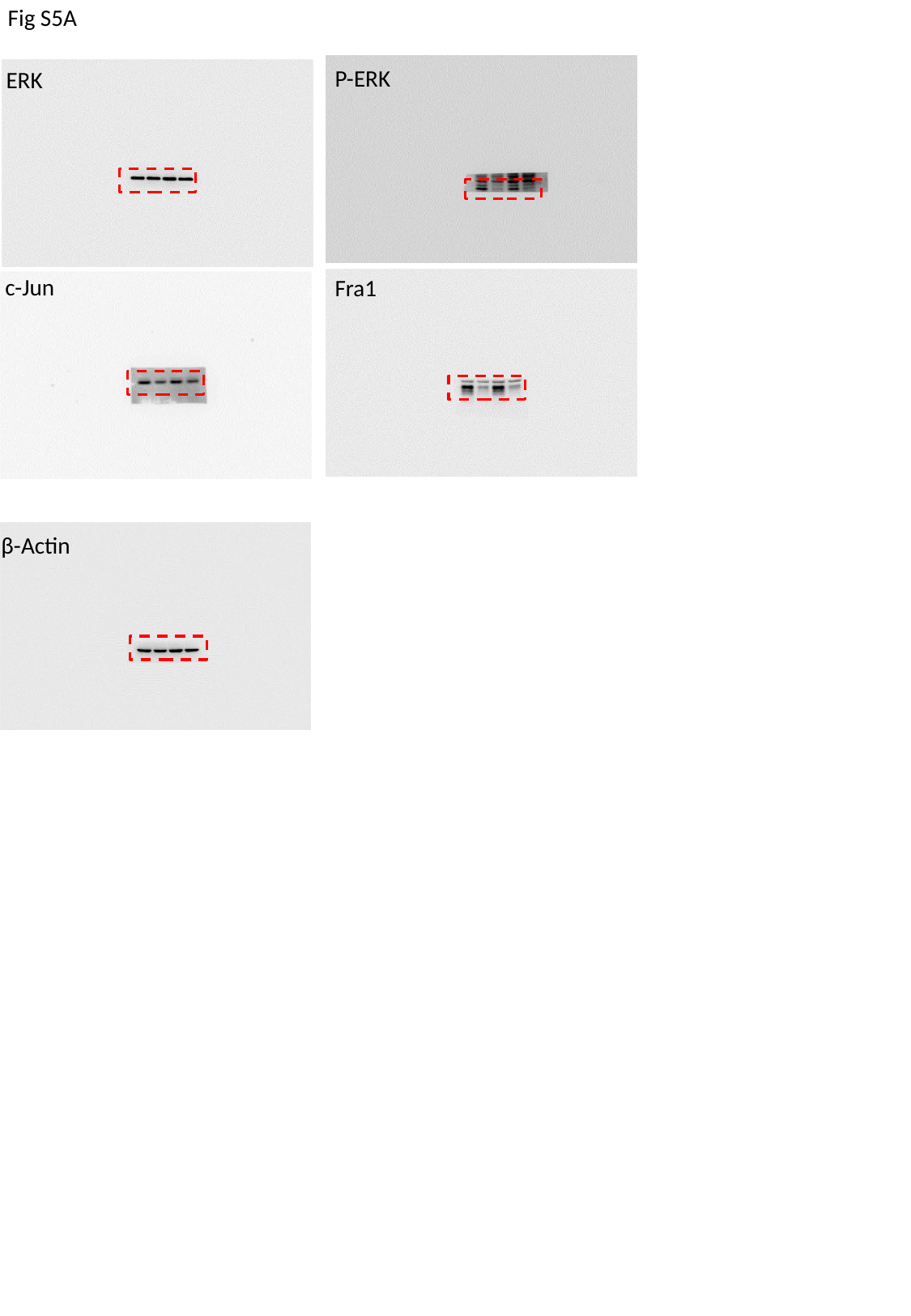

Fig S5A
P-ERK
ERK
ERK
c-Jun
Fra1
Fra1
β-Actin

## Slide 2
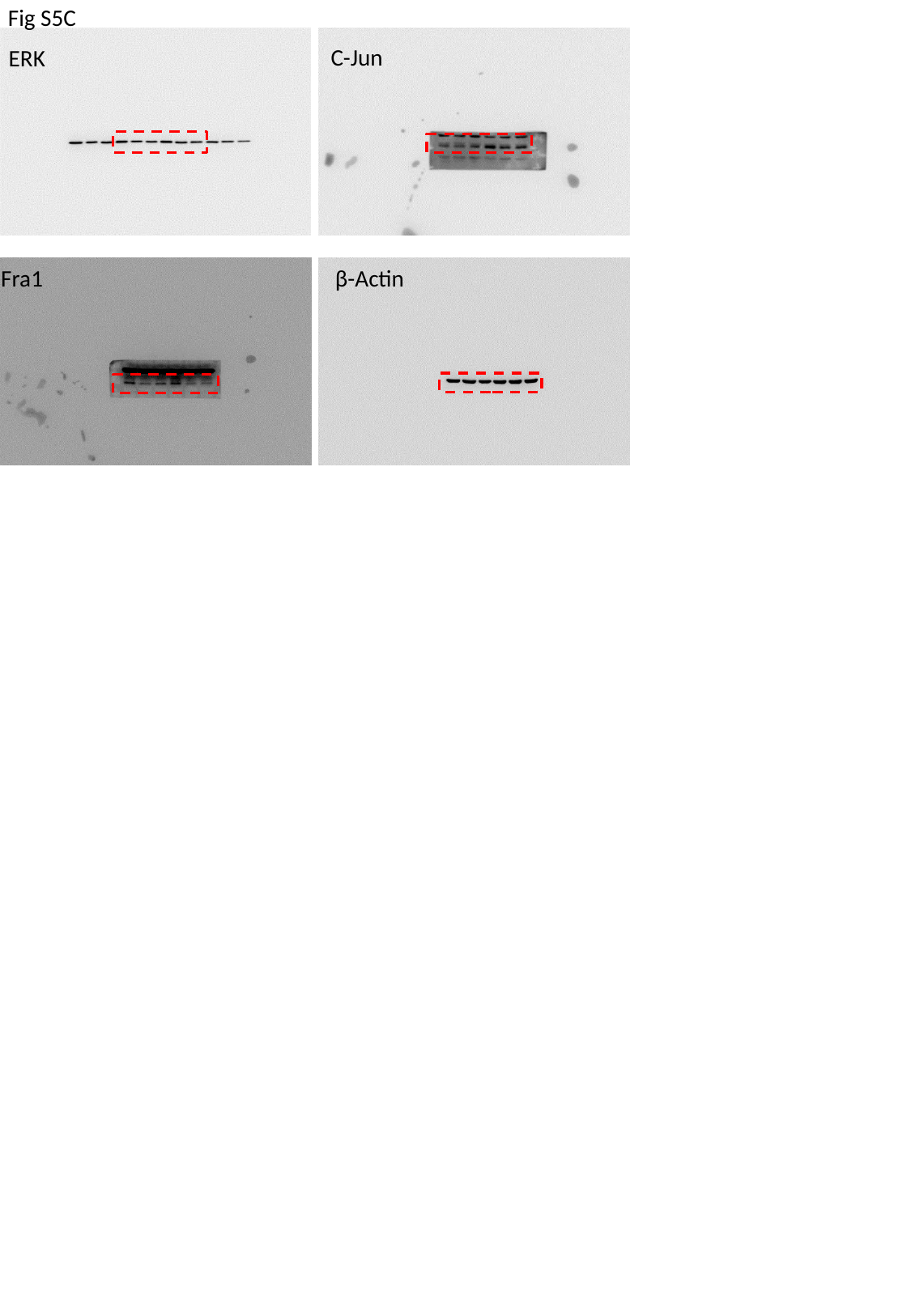

Fig S5C
C-Jun
ERK
β-Actin
Fra1

## Slide 3
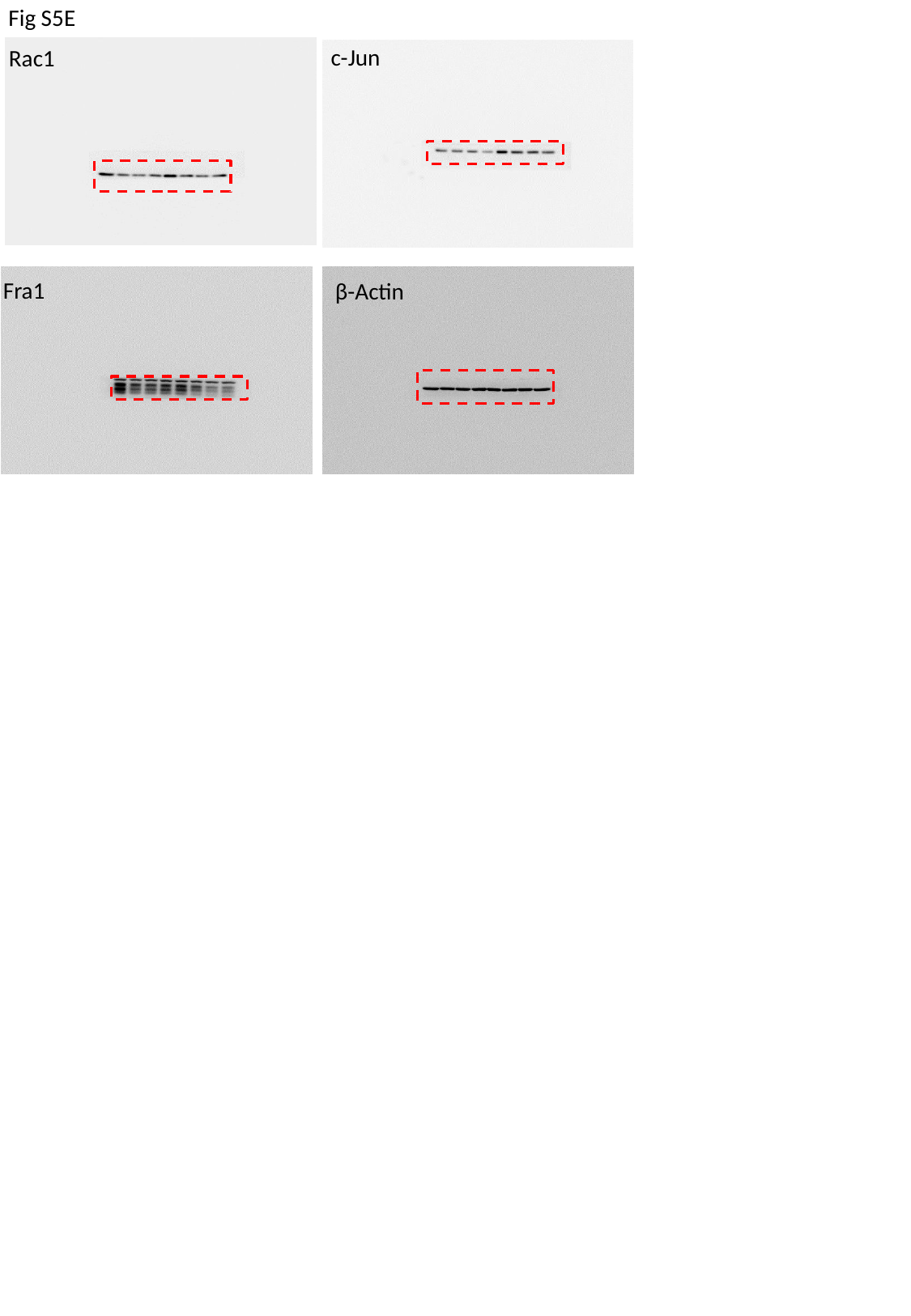

Fig S5E
c-Jun
Rac1
Fra1
β-Actin

## Slide 4
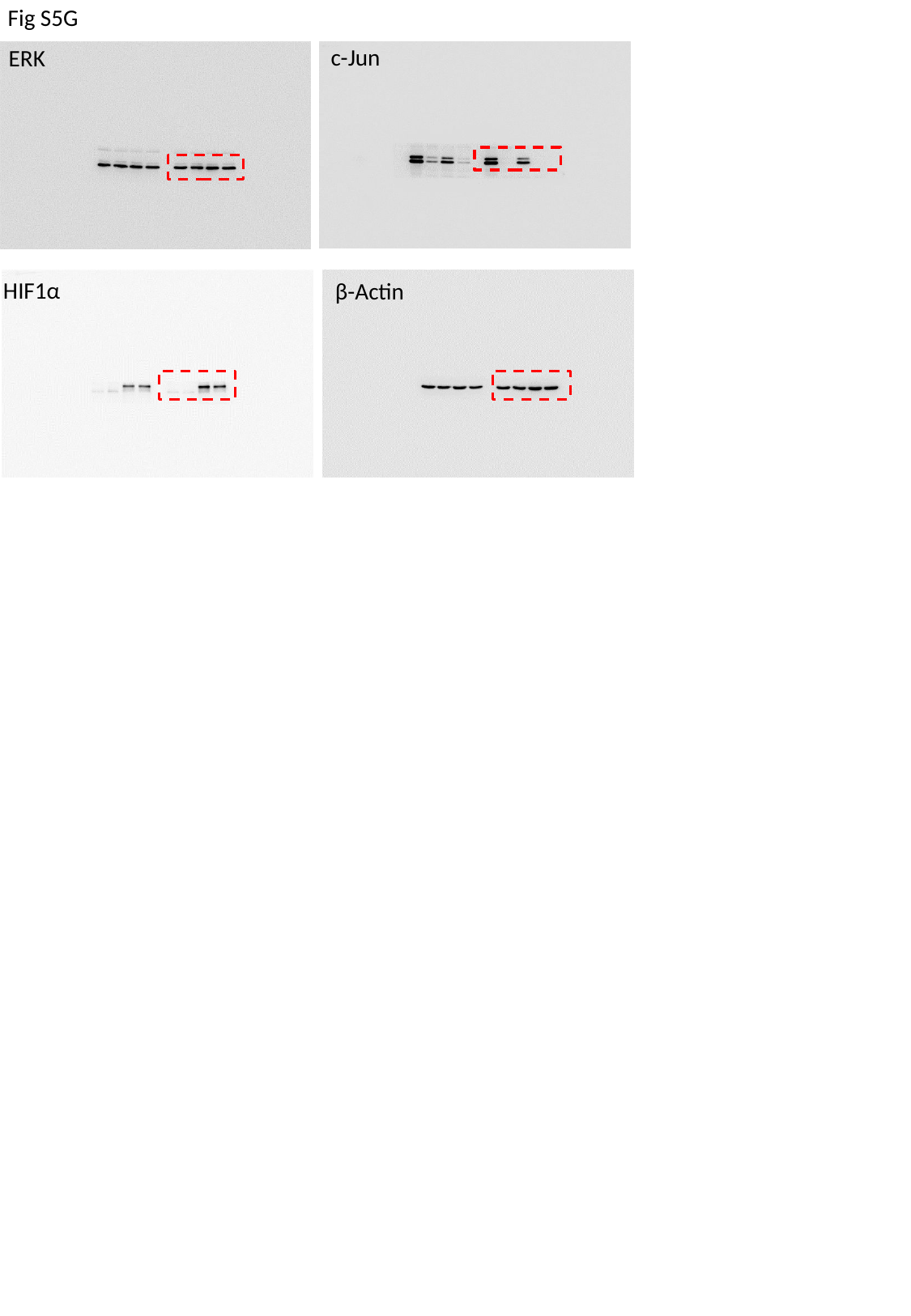

Fig S5G
c-Jun
ERK
HIF1α
β-Actin
