# Supplementary figures and images for: TNFAIP2 promotes HIF1α transcription and breast cancer angiogenesis by activating the Rac1-ERK-AP1 signaling axis
Source: Cell Death Dis. 2024 Nov 13;15(11):821. doi: 10.1038/s41419-024-07223-2 (PMC11557851; doi:10.1038/s41419-024-07223-2)

## Slide 1
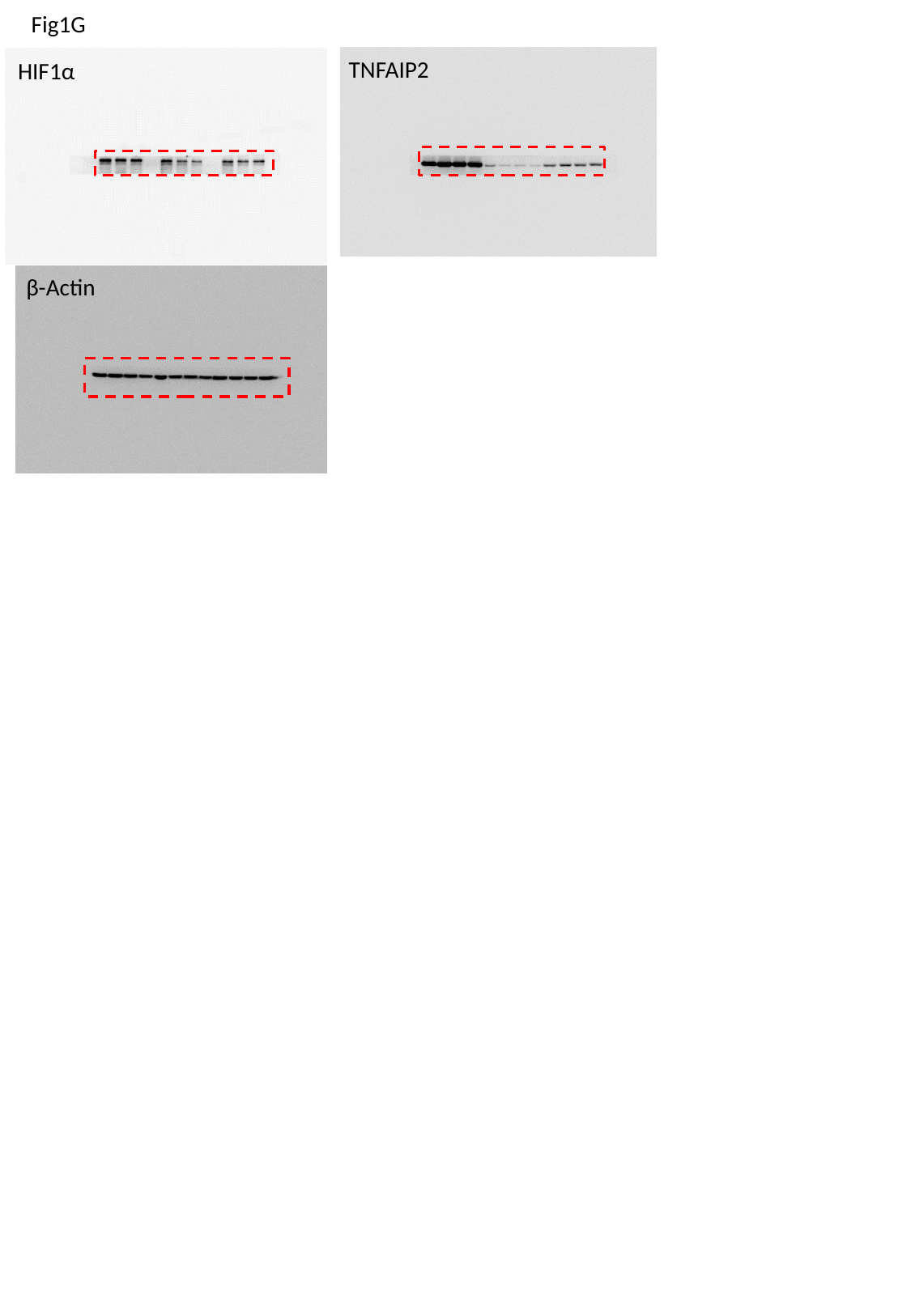

Fig1G
TNFAIP2
HIF1α
β-Actin

Supplement: Supplementary file 2 — Original western blots [file 41419_2024_7223_MOESM2_ESM.zip › Figure 1.pptx]

## Slide 1
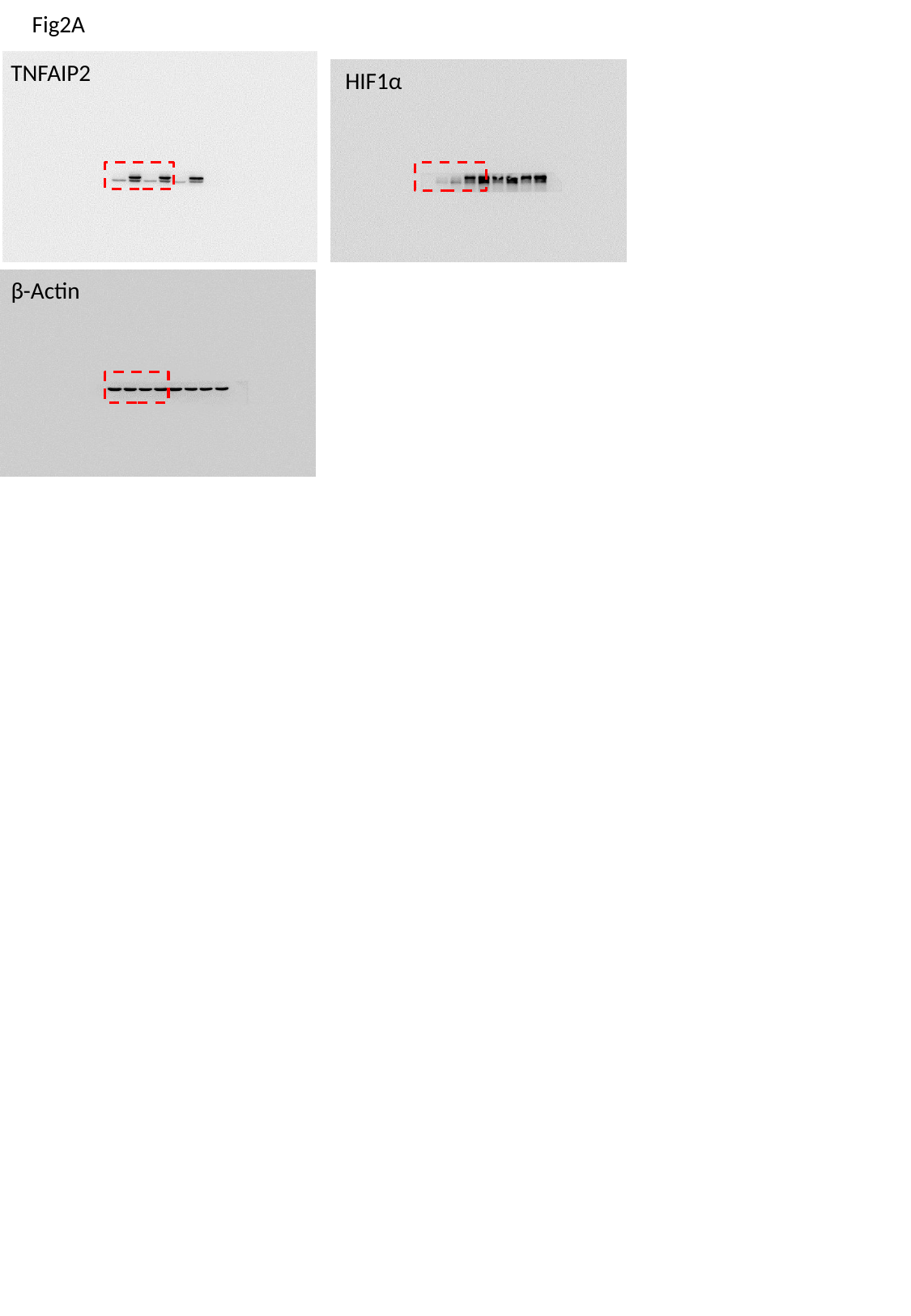

Fig2A
TNFAIP2
HIF1α
β-Actin

## Slide 2
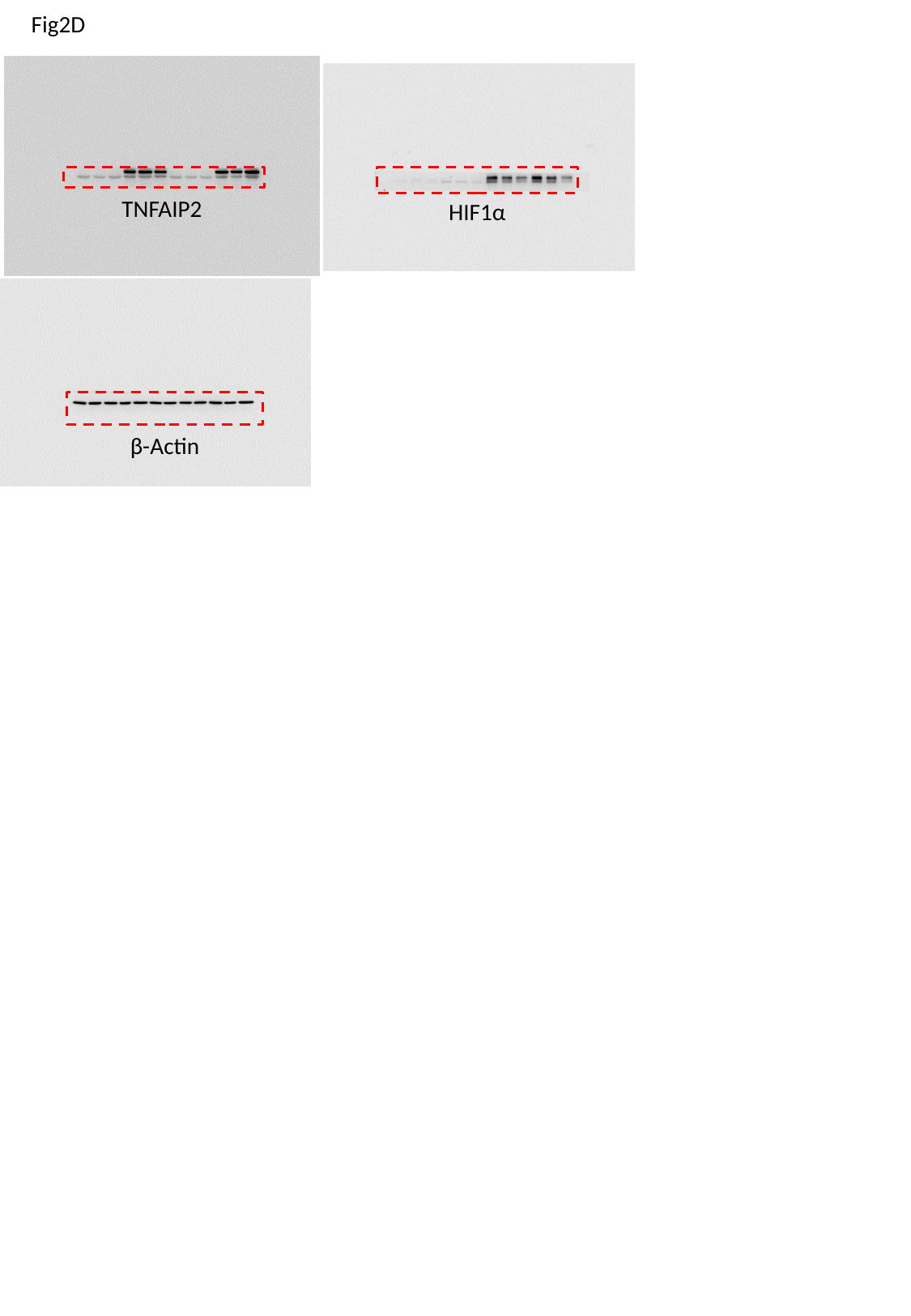

Fig2D
TNFAIP2
HIF1α
β-Actin

Supplement: Supplementary file 2 — Original western blots [file 41419_2024_7223_MOESM2_ESM.zip › Figure 2.pptx]

## Slide 1
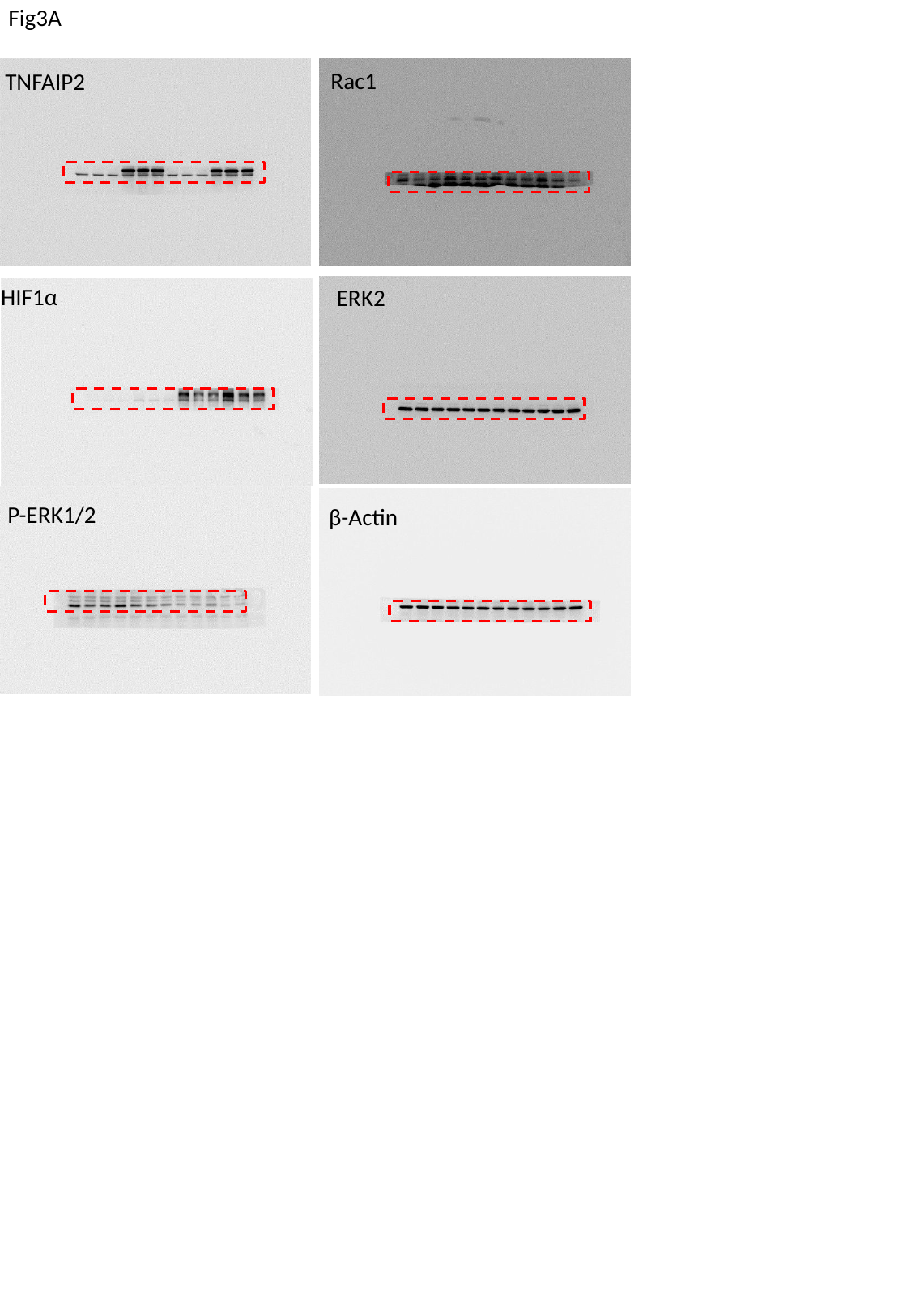

Fig3A
Rac1
TNFAIP2
HIF1α
ERK2
P-ERK1/2
β-Actin

Supplement: Supplementary file 2 — Original western blots [file 41419_2024_7223_MOESM2_ESM.zip › Figure 3.pptx]

## Slide 1
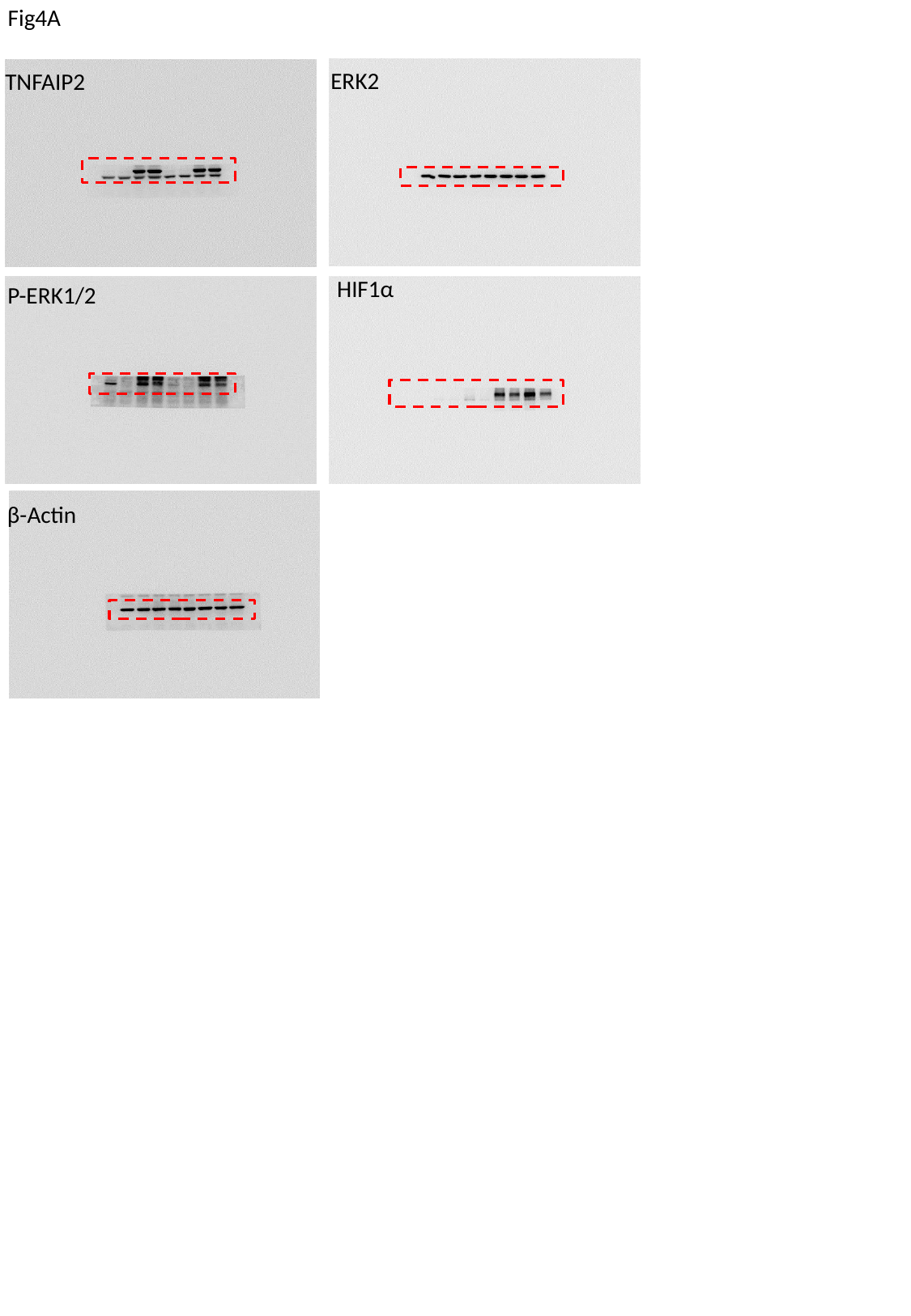

Fig4A
ERK2
TNFAIP2
HIF1α
P-ERK1/2
β-Actin

## Slide 2
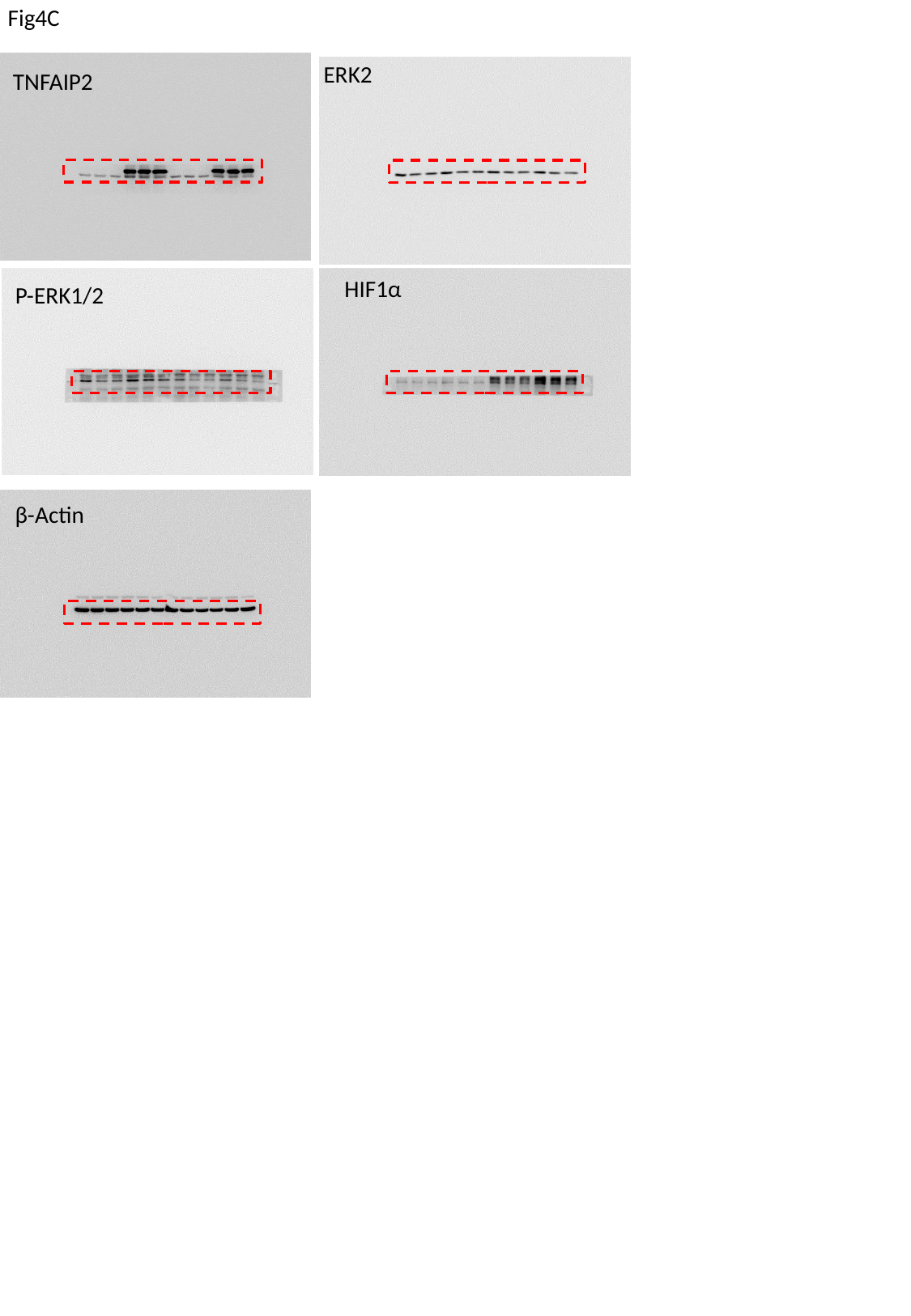

Fig4C
ERK2
TNFAIP2
HIF1α
P-ERK1/2
β-Actin

Supplement: Supplementary file 2 — Original western blots [file 41419_2024_7223_MOESM2_ESM.zip › Figure 4.pptx]

## Slide 1
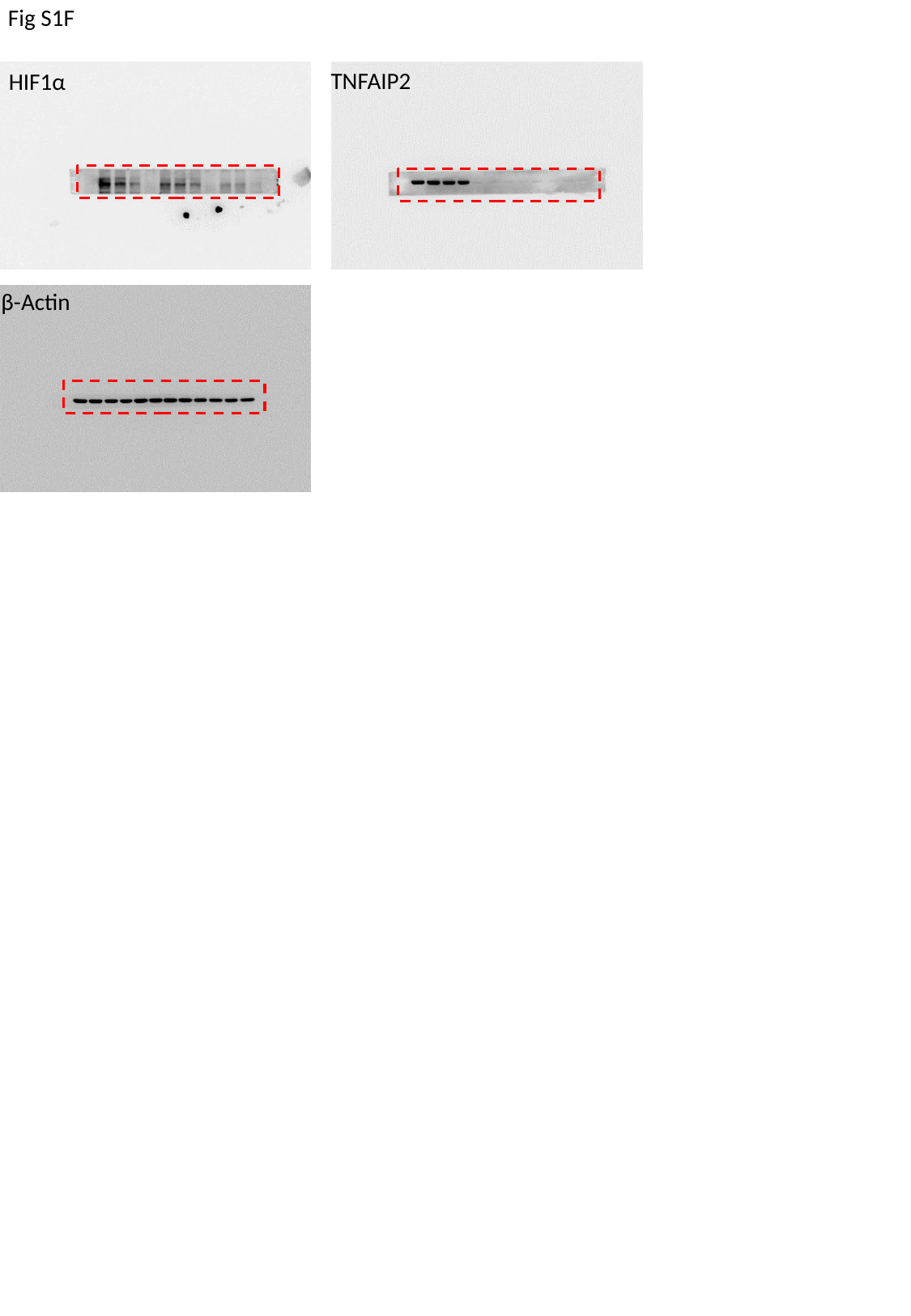

Fig S1F
TNFAIP2
HIF1α
β-Actin

Supplement: Supplementary file 2 — Original western blots [file 41419_2024_7223_MOESM2_ESM.zip › Figure S1.pptx]
